# Jet-induced rainfall seasonality and C_4_ migration over East Asia

**DOI:** 10.1038/s41467-026-74312-5

**Published:** 2026-06-10

**Authors:** Jiawei Da, Chijun Sun, Lily Serach, Timothy Gallagher, Huayu Lu, Katharine Huntington, Ran Feng, Hanzhi Zhang, Hanlin Wang, Shunchuan Ji, Zachary Sharp, Junfeng Ji, Daniel O. Breecker

**Affiliations:** 1 Department of Earth and Planetary Sciences, Jackson School of Geosciences, The University of Texas at Austin, Austin, TX USA; 2 Key Laboratory of Surficial Geochemistry, Ministry of Education, School of Earth Sciences and Engineering, Nanjing University, Nanjing, China; 3 Department of Earth and Planetary Sciences, University of California Davis, Davis, CA USA; 4 Department of Earth Sciences, Kent State University, Kent, OH USA; 5 School of Geography and Ocean Science, Nanjing University, Nanjing, China; 6 Department of Earth and Space Sciences, University of Washington, Seattle, WA USA; 7 Department of Earth Sciences, College of Liberal Arts and Sciences, University of Connecticut, Mansfield, CT USA; 8 Nanjing Institute of Geology and Paleontology, Chinese Academy of Sciences, Nanjing, China; 9 Yunnan Key Laboratory of Plateau Geographical Processes and Environmental Changes, Department of Geography, Yunnan Normal University, Kunming, China; 10 Department of Earth and Planetary Sciences, The Center for Stable Isotopes, University of New Mexico, Albuquerque, New Mexico USA; 11 Present Address: Department of Geosciences, The Pennsylvania State University, University Park, PA USA

**Keywords:** Palaeoclimate, Hydrology, Palaeoecology

## Abstract

The Neogene expansion of C_4_ grasslands transformed terrestrial ecosystems with marked influence on mammalian evolution, including hominins. However, the asynchronous C_4_ expansion on different continents makes it difficult to identify the environmental drivers, especially for higher latitudes. Here we show that rainfall seasonality governed extratropical Plio-Pleistocene C_4_ distributions in East Asia. Rainfall oxygen isotope ratios and clumped isotope soil temperatures exhibit coupled variations on the Chinese Loess Plateau (CLP) from 7 to 2.5 million years ago, indicating more spring rain during warmer times when the subtropical westerly jet was further poleward, and more concentrated summer rain under cooler climates. We attribute these changes to meridional shifts of a summer rain band on orbital and longer timescales. The most C_4_-rich ecosystems, as identified by organic carbon δ^13^C records, tracked this summer rain band, eventually eclipsing the southern CLP margin during the late Pleistocene cooling. Our model refines the East Asian paleomonsoon concept and explains the equatorward migration of extratropical C_4_ ecosystems, highlighting the tight coupling between regional rainfall seasonality and vegetation.

## Abstract (web summary)

Shifts in the westerly jet stream drove the migration of C_4_ plants across East Asia over the past 7 million years by altering rainfall seasonality, with cooler climates pushing summer rains and consequently C_4_-rich ecosystems progressively southward.

## Introduction

**Fig. 1:**
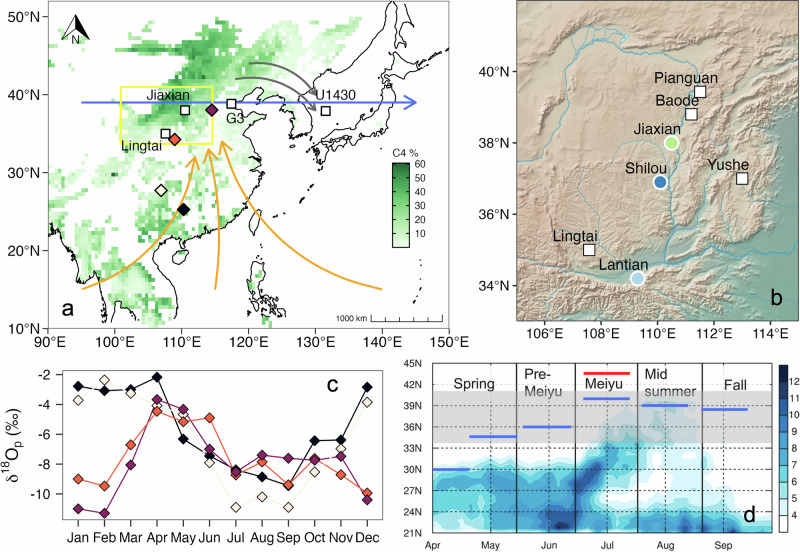
Study region maps and precipitation. **a** Modern natural C_4_ grass coverage over East Asia^87^. Open squares and colored diamonds show locations of organic δ^13^C records mentioned in this study^9–11,60^ and the modern rainfall δ^18^O data in (**c**), respectively. Grey, blue, and orange arrows show schematically the spring cold air outbreaks that deliver dust to the Japan Sea, the mean summer position of the subtropical westerly jet^88^, and the low-level southerly monsoonal flow. The yellow box shows the Chinese Loess Plateau (CLP). **b** Shaded relief map of the CLP. Circles and squares show sampling sites of this study and from literature mentioned here, respectively. Both (**a** and **b**) were plotted using the ggplot2 package in R version 4.5.0^89,90^. **c** The monthly rainfall-δ^18^O of the GNIP (Global Network of Isotopes in Precipitation) sites across the Asian monsoon region, with each site color-coded to match its location shown in (**a**). **d** Hovmöller diagram of the precipitation climatology (unit: mm/day) between 110°E and 120°E modified from ref. ^12^. The blue (modern)^88^ and red (estimated Pliocene)^35^ horizontal lines mark the seasonal migration of the westerly jet. The grey shade shows the latitudinal position of the CLP.

Growing evidence suggests that the late Neogene C_4_ expansion occurred asynchronously across different continents^1^, from as early as ~21 million years ago (Ma) in eastern Africa^2^ and 8–6 Ma on the India subcontinent^3^ to 3.5 Ma in Australia^4^. Although decreasing atmospheric CO_2_ concentrations may have set the proverbial stage^5^, this demonstrable global asynchroneity of C_4_ expansion requires regional drivers or thresholds, which are particularly poorly understood in mid-latitude regions such as East Asia (Fig. 1).

East Asian records based mainly on the δ^13^C of tooth enamel and soil carbonates (δ^13^C_c_) from the Chinese Loess Plateau (CLP) and surrounding regions, suggest C_4_ expansion during the late Miocene (~6 Ma)^6^. However, neither of these proxies provide quantitative estimates of regional C_4_ abundance. Specifically, tooth enamel may more closely record presence/absence rather than C_4_ abundance in an ecosystem^7^, and in arid-to-semi-arid ecosystems such as the CLP, soil carbonates record a mixed signal of biomass-δ^13^C, atmospheric CO_2_, and productivity^8^.

**Fig. 2:**
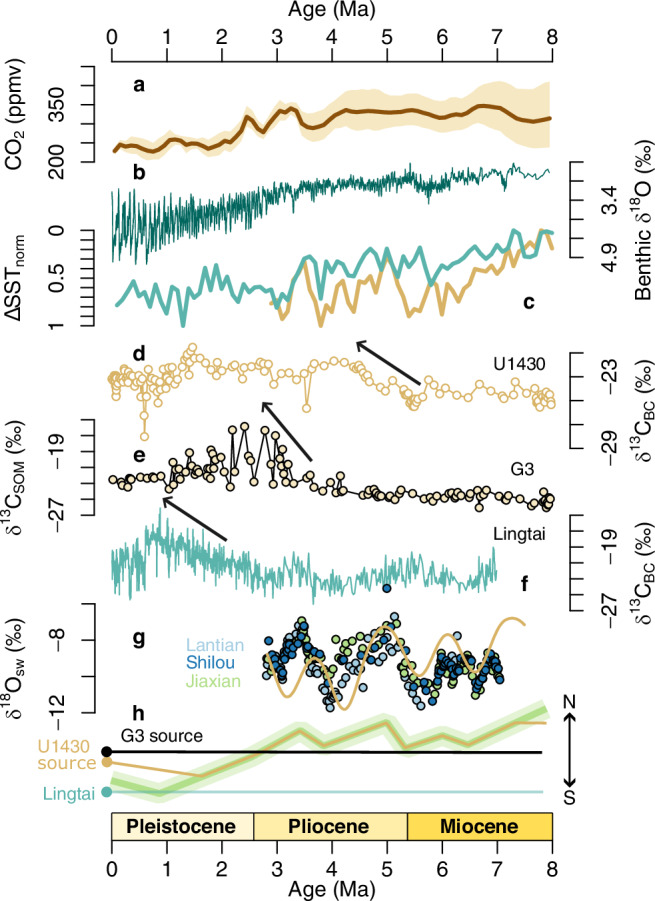
The long-term evolution of C_4_ biomass over East Asia and global climate. **a** Atmospheric CO_2_ estimates based on published proxy records via Bayesian inversion^5^. The dark line and shade show median and 68% credible interval, respectively. **b** LR04 benthic δ^18^O stack^91^. **c** Normalized meridional sea surface temperature gradient (ΔSST_norm_) based on the differences of compiled SST records from tropical and mid-latitude oceans (green curve) and from tropical and high-latitude oceans^27^ (brown curve, see “Methods”, Fig. S1). **d** Black carbon δ^13^C from IODP (Integrated Ocean Drilling Program) U1430 in the Japan Sea^11^. **e** Sediment organic matter δ^13^C from the G3 drilling core on the North China Plain^9^. **f** Black carbon δ^13^C from Lingtai section at southern CLP^10^. Arrows in (**d**–**f**) mark the timing of C_4_ expansion. **g** Reconstructed δ^18^O values of soil water (δ^18^O_sw_) from this study, color-coded by sampling sites. **h** Schematic diagram showing the meridional migration of the summer position of the westerly jet stream, drawn to be consistent with meridional temperature gradient (MTG) changes inferred from benthic δ^18^O and ΔSST_norm_. The oscillations in CLP (Chinese Loess Plateau) δ^18^O_sw_ (**g**) and the various organic carbon δ^13^C records (**d**–**f**) can be understood as responses to changes in rainfall seasonality controlled by the movement of the westerly-jet tracking summer rain band.

In contrast, soil organic matter—a more direct indicator of biomass δ^13^C—suggests that C_4_ expansion across East Asia was spatiotemporally heterogeneous (Fig. 2d–f). Consequently, multiple drivers of C_4_ expansion have been proposed, including decreasing atmospheric CO_2_ levels^9^, increased warm season precipitation^6^, intensified fire activity^10^, and enhanced long-term aridity^11^. Importantly, studies on the CLP reveal hominin occupation as early as 2.1 Ma and a close coupling between hominin migration and regional environmental conditions^12,13^. Understanding the spatiotemporal shifting of C₄-rich biomes in East Asia is thus critical for not only elucidating vegetation–climate feedbacks but also for contextualizing the evolution of hominins, which preferred C_3_ woody plants for shade, shelter, and nutrition^14^.

The regional climate over East Asia is regulated by the East Asian monsoon system (EAM). The EAM is unique owing to the dynamic interaction between the low-level, southerly monsoonal flow and the upper-level westerly jet impinging on the Tibetan Plateau^15^ (Fig. 1a). Unlike classic tropical monsoons featuring one prolonged rainfall stage, the EAM is characterized by several quasi-stationary rainfall stages, resulting from the seasonal poleward shift of the westerly jet during boreal summer (Fig. 1d)^16^. Mounting evidence from paleoclimate records and climate models underscores the role of this jet transition in controlling the meridional migration of the monsoonal rain band, and consequently rainfall seasonality over East Asia on timescales from millennia to glacial cycles^15,17^. However, the role of the jet transition in rainfall seasonality over longer timescales remains unknown.

We hypothesized that the asynchronous C_4_ expansion and retreat across East Asia was paced by shifting warm season precipitation in response to the long-term equatorward migration of the westerly jet during the Plio-Pleistocene global cooling. To test this hypothesis, we reconstructed δ^18^O values of the late Miocene-Pliocene rainfall (δ^18^O_p_) over the CLP as an independent test of long-term, jet-transition-driven changes in East Asian rainfall seasonality. We also evaluated, by considering paleoclimate simulations, whether the concept of jet-transition-modulated changes in rainfall seasonality is plausible and, by comparison with previously published paleoenvironmental records, whether our hypothesized mechanism is broadly explanatory.

## Results and discussion

### Coupled T_Δ47_–δ^18^O_p_ time series

**Fig. 3:**
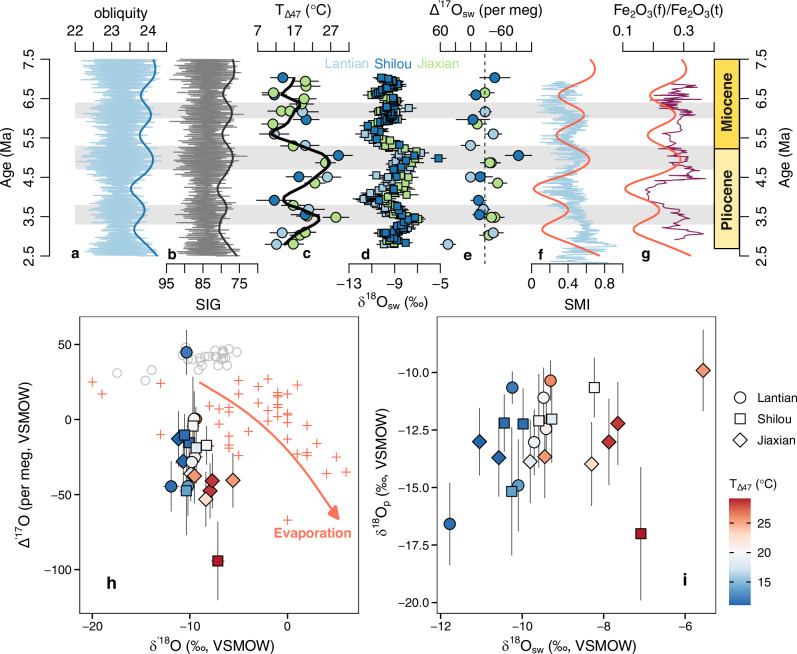
Reconstructed soil-water isotopic compositions. **a**, **b** Obliquity signal in La2004 astronomical solution and 25°N–65°N summer insolation gradient (SIG) along with their filtered ∼1.2-Myr amplitude modulation cycles, respectively. **c–e** Time series of the reconstructed T_Δ47_, δ^18^O_sw_, and Δ^‘17^O_sw_, color-coded by sampling sites. Calcite formation temperatures used to calculate δ^18^O_sw_ were obtained by interpolating T_Δ47_ values within individual sections. Interpolation was restricted to intervals bracketed by adjacent T_Δ47_, and the resulting temperature uncertainty was propagated into the δ^18^O_sw_ estimates. δ^18^O_sw_ calculated from paired δ^18^O_c_-T_Δ47_ measurements show similar variations across sections (Fig. S2). The Δ′^17^O of soil waters (Δ′^17^O_sw_) were calculated from paired T_Δ47_-Δ′^17^O_c_ measurements (“Methods”). Error bars and the black line in (**c**) show 1σ errors from replicate analyses and the LOESS line (span = 0.3), respectively. Note the reversed y-scales for SIG and Δ′^17^O_sw_. **f** Summer monsoon index (SMI) based on soil magnetic susceptibility and carbonate content from Lingtai section^14^. **g** Free iron to total iron ratio from Pianguan section^92^. The red curves in (**f**, **g**) show the 1.2-Myr obliquity amplitude modulation cycle. **h** Reconstructed Red Clay soil waters compared with global Holocene soil waters^19^ (red crosses) and modern meteoric waters across China^18^ (grey circles). The red arrow highlights the evaporation trend seen in Holocene soil waters. **i** Posterior estimates of δ^18^O_p_ using the Bayesian inversion of a soil water isotope model plotted against δ^18^O_sw_ (see “Methods”). Error bars in (**d**, **e**, and **h**) extend to the 16th and 84th percentiles of distributions propagated using Monte Carlo random sampling, whereas those in (**i**) extend to the 16th and 84th percentiles of the modeled posterior distributions. Red Clay data in (**h** and **i**) are color-coded by T_Δ47_ and shape-coded by site.

We reconstructed rainfall δ^18^O_p_ using the clumped isotope (Δ_47_) and triple oxygen isotope compositions of calcite nodules collected from three Red Clay sections (Lantian, Shilou, and Jiaxian) on the CLP (Fig. 1b, see “Methods”). Although replication per sample is limited (*n* = 2–6) by material availability, analytical uncertainties are fully propagated, and interpretations are based on consistent patterns across the dataset rather than individual temperature estimates. The clumped isotope temperature (T_Δ47_) and δ^18^O_sw_ from these three sections on the CLP show generally coherent trends across the late Miocene–Pliocene (Fig. 3c, d).

In addition to surface temperature, changes in pedogenic carbonate formation depth may contribute to T_Δ47_ variations. Nonetheless, modeled monthly soil temperatures at potential carbonate formation depths vary by less than 3.3 °C based on modern observations across the CLP (Fig. S3), indicating the minor effect of carbonate formation depth on T_Δ47_ compared to seasonal surface temperature variations.

Overall, both T_Δ47_ and δ^18^O_sw_ are higher during the Pliocene (5.3–2.7 Ma), with mean values of 20 °C (SD = 5 °C, *n* = 19) and −8.9‰ (SD = 1.1‰, *n* = 139), respectively, compared to the late Miocene (7–5.3 Ma) with lower averages of 16 °C (SD = 4 °C, *n* = 15) and -9.7‰ (SD = 0.6‰, *n* = 105). Superimposed on this long-term trend are three pronounced peaks in both T_Δ47_ and δ^18^O_sw_. The highest peak occurred at 5.1 Ma spanning the Miocene–Pliocene boundary (MPB, Fig. 3), with values of 29 ± 4 °C and −6.7 ± 0.7‰, accompanied by two smaller peaks at 6.2 Ma and 3.5 Ma.

The Δ′^17^O_sw_ values show large fluctuations ranging between −94 ± 26 per meg at 5.1 Ma and 45 ± 15 per meg at 2.8 Ma (Fig. 3e), with a mean value of −27 per meg (SD = 27 per meg, *n* = 22). The Δ′^17^O_sw_ are significantly lower than those of modern meteoric waters across China^18^ but generally overlap with those of soil waters calculated using a global Δ_47_–Δ′^17^O dataset of Holocene pedogenic carbonates^19^ (Fig. 3h). However, there is no coherent trend in Δ′^17^O_sw_ through time (Fig. 3e) or in Δ′^17^O_sw_–δ^18^O_sw_ space (Fig. 3h), indicating that the observed δ^18^O_sw_ variability is not primarily driven by evaporation. Moreover, posterior δ^18^O_p_ estimates from the soil-water-isotope model reveal a generally positive correlation with δ^18^O_sw_ (Fig. 3i), indicating that δ^18^O_sw_ variations are largely explained by increases in δ^18^O_p_ with warming and decreases with cooling.

### Westerly Jet-induced rainfall seasonality

In the EAM region, δ^18^O_p_ records are typically interpreted to record rainout, with higher δ^18^O_p_ corresponding to a weaker monsoon circulation and a drier climate associated with less rainfall upstream and/or less summer rainfall locally^20,21^. We propose that instead of annual rainfall amount or the overall EAM strength, our observed δ^18^O_p_ variations record changes in rainfall seasonality closely linked to the westerly jet stream dynamics.

**Fig. 4:**
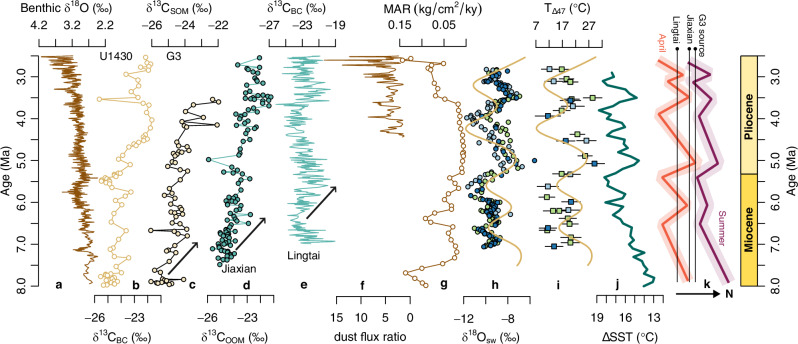
The late Miocene–Pliocene evolution of the westerly jet, C_4_ biomass, and global climate. **a** LR04 benthic δ^18^O stack^91^. **b–e** Organic carbon δ^13^C records across East Asia, including black carbon from U1430^11^, sediment organic matter from the G3 drilling core^9^, soil organic matter δ^13^C from Jiaxian section^60^, and black carbon from Lingtai section^10^. Note G3 data after 3.5 Ma are outside the axis range and therefore not shown here. **f** Dust flux ratio between ODP sites 1208 and 885/886 from the mid-latitude North Pacific Ocean^22^. **g** Dust flux record from ODP site 885/886^25^. **h**, **i** Reconstructed δ^18^O_sw_ and T_Δ47_ from this study. **j** Meridional sea surface temperature gradient (ΔSST) based on compiled SST records from tropical and high-latitude oceans (see “Methods”, Fig. S1). **k** Schematic diagram showing the meridional migration of the jet position during summer and April with respect to sampling locations of δ^13^C records, drawn to be consistent with MTG changes inferred from ΔSST. The yellow curves in (**f**–**h**) show the 1.2-Myr obliquity amplitude modulation cycle.

Under warmer climates, such as the beginning of the Pliocene, the reduced MTG (Fig. 4j) resulted in the weakening and northward migration of the westerly jet^22^ (Fig. 4k), leading to a seasonally earlier onset (spring rather than summer) of rainfall over northern China^15^ and thus higher δ^18^O_sw_ values (Fig. 4h). Moreover, the weakened jet impinging upstream on the Tibetan Plateau caused a weaker stationary Rossby wave downstream, resulting in high pressure anomalies over East Asia and the northward shift of the North Pacific subtropical high^23^. Consequently, the southerly wind along the northwestern edge of the subtropical high migrated northward, resulting in an earlier onset of spring rainfall.

The modern monthly δ^18^O_p_ pattern across the EAM region is characterized by the transition from the higher-δ^18^O spring to lower-δ^18^O summer rain, primarily controlled by distillation upstream^21^ (Fig. 1c). Because the seasonal march of the monsoonal rain was already established during the Miocene^24^, it is reasonable to assume a quasi-stationary monthly δ^18^O_p_ climatology through time. The modern spring-to-summer δ^18^O_p_ shift exceeds 5‰ (Fig. 1c), comparable to changes in the reconstructed δ^18^O_sw_ (Fig. 2g). Thus, the tight T_Δ47_–δ^18^O_sw_ coupling (*p*-value < 0.001, Fig.e S4) supports rainfall seasonality changes as the main driver of the observed δ^18^O_sw_ variability, with more spring rain (high δ^18^O_p_) during warmer times when the westerly jet was positioned at a higher latitude.

Several lines of evidence support the MTG—jet—rainfall seasonality mechanism as the driver of coupled T_Δ47_–δ^18^O_p_ variations. First, the effect of the MTG on jet position and strength has been documented by dust flux and provenance records from the North Pacific Ocean^22,25,26^ (Fig. 4f, g), suggesting poleward and weakened westerly winds during the warm Pliocene compared with the late Miocene and Pleistocene glacial periods. Prominent maxima in our observed T_Δ47_ and δ^18^O_sw_ correspond well with minima in meridional sea surface temperature (SST) gradients^27^ at 5.0 and 3.5 Ma (Fig. 4h–j), thereby linking temperature and rainfall with the westerly jet position through the MTG.

Moreover, our T_Δ47_ records show large amplitude of variations (>10 °C) across the MPB (Fig. 3c). For comparison, mean annual surface temperature (MAST) variability on the CLP across the Pleistocene glacial cycles is less than 10 °C^28^. Given that regional climate variability is much less pronounced during the late Miocene–Pliocene periods^29^, it is unlikely that changes in T_Δ47_ are solely caused by MAST. Pedogenic carbonates tend to record seasonal climate signals rather than annual means^30^. In arid regions such as the CLP, pedogenic carbonate formation is primarily driven by rainfall events through its control over soil water chemistry^31^. Consequently, pedogenic carbonates grow after the rainy season under enhanced evaporation and reduced soil respiration^32^. During cold periods, CLP rainfall occurred during summer due to the lower latitude position of the westerly jet. Consequently, pedogenic carbonates grew during late summer and early fall, recording low summer-δ^18^O_p_ and fall temperature. During warmer periods, an early onset of spring rain and intensive summer evaporation likely facilitate pedogenic carbonate growth during mid-summer, recording high spring-δ^18^O_p_ and summer temperature. Modern surface temperature difference on the CLP between July and October can be as high as 17 °C. Thus, the large T_Δ47_ variability we observe is likely caused by seasonal shifts in soil carbonate formation superimposed on the long-term MAST changes.

The jet transition hypothesis is also supported by model simulations. Specifically, we looked at three experiments, mid-Pliocene 280 ppm (Eoi280) and 400 ppm (Eoi400), and pre-industrial control (E280), from the Community Earth System Model version 2 (CESM2), following the Pliocene Model Intercomparison Project phase 2 (PMIP2)^33,34^ (See “Methods”). Both Eoi280 and Eoi400 show a weakening and northward shift of the westerly jet compared to E280 (Fig. S5a, b), which led to a northward shift of the western Pacific subtropical high and an earlier seasonal march of the EAM (Fig. S5c, d). These shifts simulated using CESM2 are consistent with the PMIP2 multi-model ensemble mean^35^. Consequently, northern China (>30°N) received more spring rain and less summer rain during the warm Pliocene compared to the pre-industrial conditions (Fig. S5e, f).

Westerly jet-related east Asian rainfall seasonality broadly reconciles discrepancies among various proxy records. For instance, soil magnetic susceptibility suggest long-term wetting trend across the Pliocene^36^, whereas land snail species^37^ and organic biomarkers^38^ indicate the opposite (Fig. S6). Magnetic susceptibility is mainly controlled by the abundance of ultrafine ferrimagnetic particles^39^ formed during redox potential changes caused by soil wet-dry cycles^40^, which are most prominent during the summer when potential evapotranspiration is large and soil respiration is water-limited^41^. In this view, increases in magnetic susceptibility toward the Pliocene-Pleistocene boundary^36^ (Fig. S6b) were caused by larger or more frequent wet-dry cycles as monsoonal rainfall seasonality shifted from spring to summer. This seasonality shift also explains the terrestrial snail record (Fig. S6e), which shows a dominance of warm-humid-adapted species during the early Pliocene. Because land snails tend to be active at high relative humidity (RH) (>70%) and intermediate temperature (10–27 °C)^42^, spring rain during the early Pliocene resulted in greater water availability due to lower evapotranspiration, which favored the growth of humid-loving taxa. On the other hand, soil bacteria likely reached peak metabolism during the summer growing season^43,44^, and thus the soil tetraether proxy likely records summer drought during the warm early Pliocene (Fig. S6c).

The jet-induced rainfall seasonality also resolves a long standing perplexity. During the Pliocene, rainfall maxima over East Asia occurred at obliquity minima as recorded by magnetic susceptibility (a proxy of wet-dry cycles) from the CLP and marine proxies related to runoff (e.g., salinity, productivity, lithogenic accumulation) from the South China Sea^45^. This relationship is the opposite of that expected from the effects of fast physics on monsoonal rainfall. For example, at obliquity minima, northern hemisphere summer insolation is small, which should result in smaller land-sea and cross-equatorial pressure gradients and thus weaker monsoonal circulation. Therefore, in the established framework of the EAM, the observed rainfall phasing at the obliquity band has been enigmatic^45^. However, if we view the EAM from the perspective of the jet transition, there is a dynamic explanation for rainfall maxima at obliquity minima. During the obliquity maxima, the mean position of the westerly jet was further north, which pushed summer rainfall maxima to a higher latitude (north of Jiaxian according to our records), and consequently, resulted in fewer summer wet-dry cycles on the CLP and less surface runoff across southern China. The zone of intense summer rainfall moved southward at obliquity minima, following the mean position of the westerly jet.

### Westerly Jet-induced C_4_ migration

The meridional migration of the westerly jet provides a dynamic explanation for the asynchronous C_4_ expansion in East Asia. Specifically, the summer jet position likely maintained a variable but generally high latitude across the late Miocene–Pliocene, resulting in rainier spring in East Asia and consequently, the dominance of C_3_ plants as indicated by the overall low organic carbon δ^13^C values (Fig. 2d–f). This is supported by model results, showing the summer jet located at >40°N under Pliocene boundary conditions (Fig. S5).

We suggest that the long-term increase in MTG since the late Miocene (Fig. 2c) gradually pushed the mean jet position (Fig. 2h), and thus the summer rain band, equatorward, causing the north to south migration of C_4_-rich biome. Organic carbon δ^13^C values increase first (5.3–4.0 Ma) in the U1430 record from the Japan Sea (Fig. 2d), consistent with modest C_4_ expansion at these high latitudes. Subsequently, the onset and intensification of the Northern Hemisphere Glaciation (NHG) pushed the mean jet position further south, as suggested by increases in dust fluxes in the mid-latitude North Pacific Ocean^22^ (Fig. 4f, g). Meanwhile, organic carbon δ^13^C values increase (3.5–2.5 Ma) and then decrease in the G3 record (Fig. 2e), consistent with the migration of a C_4_-rich biome across the North China Plain. With further cooling and high-latitude ice sheet expansion during the Pleistocene, organic carbon δ^13^C values increased (2–1 Ma) in the southernmost Lingtai section (Fig. 2f), consistent with a C_4_-rich biome reaching the southern of the CLP. The subsequent decrease towards the late Pleistocene likely reflects a northward C_4_ retreat as the climate warmed and the MTG decreased slightly (Fig. 2b, c).

Specifically, because sediments at U1430 were mainly of eolian origin brought by the spring storm outbreaks and the westerlies^11^ (Fig. 1a), they should largely originate from regions that experienced dry spring. Thus, we should expect the source region to track the summer rain band (Fig. 2h). The shifting regimes of sediment source explains both the increased δ^13^C at U1430 during 5–4 Ma despite the southward migration of the jet, and the persistently high δ^13^C from 4.2 to 1.7 Ma (Fig. 2d). Notably, the mean summer jet position likely overlapped with the dust source regions of U1430 beginning in the late Miocene (Fig. 2h), yet δ^13^C did not increase until the MPB (Fig. 2d). We attribute this discrepancy to the significant surface cooling in East Asia during 8–7 Ma^46^, which likely inhibited the growth of C_4_ plants^46^. The Pleistocene C_4_ expansion is also witnessed in organic δ^13^C records from other sections located on the southern CLP^47,48^. The long-term equatorward C_4_ migration is inconsistent with a direct control by decreasing atmospheric CO_2_ (Fig. 2a).

The jet transition also explains some of the small δ^13^C shifts across the Miocene–Pliocene. During the late Miocene cooling, as the jet shifted equatorward gradually, organic carbon δ^13^C increased by ~2‰ first in the north at G3, then at Jiaxian, and latest at Lingtai, possibly resulting from progressive, small-scale, equatorward C_4_ expansion (Fig. 4c–e). As the MTG decreased significantly with warming across the MPB (Fig. 4j), the jet and consequently summer rainfall maxima migrated poleward, leading to rainier spring and C_4_ retreat in northern China as evidenced by δ^13^C decreases at both Lingtai and Jiaxian (Fig. 4e, f). The jet shifted equatorward again during 5–4 Ma, resulting in C_4_ expansion in G3 source regions and Jiaxian, whereas Lingtai was still dominated by C_3_ plants (Fig. 4c–e). A consistent spatiotemporal pattern emerges in which warm season precipitation was the main limiting factor governing the multi-stage southward C_4_ progression across East Asia, paced by the latitudinal migration of the westerly jet.

### Obliquity-paced changes in the meridional insolation gradient

Although the late Pliocene-Pleistocene evolution of the MTG, the westerly jet, and C_4_ biomass can be explained by the NHG^49^, their variations prior to the NHG require an additional mechanism. Orbital insolation directly impacts the MTG and consequently monsoon rainfall stages^50^. The 41-ky obliquity cycle influences meridional insolation gradients by affecting high latitude climate as well as global ice volume^51^ (Fig. 3a, b). Importantly, the 1.2-Myr cycle that corresponds to the amplitude modulation of obliquity (Fig. 3a) is thought to play a critical role in glaciation and sea-level changes^52^. The MTG based on the compiled sea surface temperature records, is generally in pace with the 1.2-Myr cycle between 7 and 3 Ma (Fig. 4h–j).

Our T_Δ47_ and δ^18^O_sw_ records show clear 1.2-Myr cycle, with three δ^18^O_sw_ peaks co-occurring roughly with maximum amplitudes of the 41-kyr obliquity cycle (Fig. 3a–d). During the peak obliquity maxima, enhanced high-latitude summer insolation led to reduced meridional insolation gradient and MTG (Fig. 4j), thereby weakening the jet and pushing it further north, leading to an earlier onset of spring rain in northern China. This 1.2-Myr rhythm was also registered in other proxy-derived EAM records^53^ (Fig. 3f, g), supporting its critical role in modulating the regional water cycle.

It should be noted that the 1.2-Myr cycle controls the amplitude of the obliquity cycle (Fig. 3a), meaning both highest obliquity maxima and lowest minima occur at peak amplitudes. Our T_Δ47_ and δ^18^O_sw_ records preserve only the obliquity maxima signals, probably due to overprinting. Specifically, during periods other than obliquity maxima, the jet was at a lower latitude and monsoonal rain was more concentrated during summer, when temperature and evaporation are relatively higher than spring, leading to less soil water penetration and shallower formation of pedogenic carbonates. Whereas during obliquity maxima, early onset of monsoonal rain, cooler temperature, and less evaporation during spring allowed soil water to percolate deeper into soils, leaching previously formed shallower carbonates and resetting isotopic signals. In the northern Shilou and Jiaxian sections, the mean sedimentation rate was ~10 m/Ma^36^, corresponding to 40 cm of sediment per 41-kyr obliquity cycle. The modern mean annual precipitation (MAP) across the CLP ranges between 250 and 650 mm, which corresponds to carbonate accumulation depth of ~40–70 cm^54^. Assuming MAP during the late Miocene–Pliocene was within this range, pedogenic carbonates formed during the previous obliquity maxima should be at ~80–110 cm depth at the next maxima, which would require extremely high MAP of more than ~700–1000 mm to dissolve them. Thus, these deepest-formed carbonates were selectively preserved, recording seasonal environmental conditions during the obliquity maxima.

The MTG, δ^18^O_sw_, and the 1.2-Myr cycle fell out of phase at 4.1 Ma, when the obliquity amplitude minima preceded the MTG maxima and δ^18^O_sw_ minima (Fig. 4h, i). These offsets likely reflect the increasing influence of NHG on the MTG, the jet position, and rainfall seasonality during the Plio-Pleistocene transition. The prevalence of ice-sheet-related slow physics over insolation-driven fast physics is supported by damped orbital-scale variability of both the East Asian winter and summer monsoon as well as their out-of-phase relationship starting 4.2 Ma^29^.

Taken together, our nodule-based isotope records spanning 7–2.5 Ma provide empirical evidence supporting the jet transition hypothesis across orbital-to-tectonic timescales. Consequently, the jet transition offers a unified explanation of the multi-phase C_4_ migration across East Asia by modulating rainfall seasonality. This complex history of C_4_ migration reflects the dynamic interplay between global climate forcings (i.e., ice sheets, insolation, jet dynamics) and regional factors (i.e., EAM, topography).

## Methods

### Sampling

The CLP is located on the current northern margin of the EAM region and is sensitive to monsoonal rainfall changes^55^ (Fig. 1a). The Miocene–Pliocene deposits on the CLP, known collectively as the Red Clay formation, record nearly continuous paleoenvironmental information from 8 to 2.5 million years ago (Ma)^56^. Bulk paleosol and pedogenic carbonate nodule samples were collected from three Red Clay sections: Lantian, Shilou, and Jiaxian. The 66-m thick Lantian section (34.190°N, 109.234°E, top elevation = 630 m) was located on the southern edge of the CLP, whereas the 58-m Jiaxian (38.272°N, 110.090°E, top elevation = 1025 m) and 71.4-m thick Shilou sections (36.926°N, 110.928 °E, top elevation = 1160 m) were situated in the northern CLP (Fig. 1). Modern MAP decreases from 720 mm in the southern Lantian to 529 mm and 433 mm in the northern Shilou and Jiaxian. Mean annual air temperatures (MAT) are 10.9 °C, 10.0 °C, and 14.6 °C in Jiaxian, Shilou, and Lantian, respectively. Bulk paleosol samples were collected at 10 cm intervals, and at least two nodule samples were collected from each distinct depth. To avoid regolith contamination, soil profiles were trenched 1 m deep before sampling.

### Age model

The magnetic susceptibility data from each section were used to correlate to published age models^36,57,58^, which were established through linear interpolation using geomagnetic reversals as age control. The paleomagnetic records contain every polarity subchron spanning 7.0–2.5 Ma. The relatively stable sedimentation rate across the late Miocene–Pliocene also suggest slow and continuous deposition^36,57,58^. Because the Red Clay formation is an aggregated soil profile, it is not possible to constrain the depth of carbonate nodule formation. We therefore use the depositional ages of the soil layer where the nodules were formed to represent the ages of nodules. Based on the modern MAP across the CLP and the MAP-Bk depth relationship^59^, the depth of pedogenic carbonate formation is ~40–70 cm, which corresponds to ~20–50 ka offset given the sedimentation rate of the three Red Clay profiles. This age uncertainty is minor when interpreting long-term tectonic-scale records derived from pedogenic carbonates. Moreover, published pedogenic carbonate δ^13^C records from multiple Red Clay sections co-varied with near-in-situ proxies, including magnetic susceptibility and the δ^13^C of soil organic matter^47,60^, suggesting any potential lags due to translocation are minor at the timescale of interest here.

### Sample pretreatment

We conducted stable isotope analyses on calcite nodules (*n* = 970) collected from the three Red Clay sections at Lantian, Shilou, and Jiaxian. At least two individual nodule samples from each stratigraphic layer were used for δ^18^O analyses. Owing to budget constraints and the time-intensive nature of both analyses, we selected 32 and 22 nodules for clumped and triple oxygen isotope analyses, respectively. The primary criterion for selection was to maximize temporal coverage to capture long-term trends and variations. Sampled materials are not subject to solid-state reordering of clumped isotope signals due to the shallow burial depth (<100 m). Prior to analyses, calcite nodule samples were either ultrasonically rinsed and then oven-dried at 50 °C overnight or physically cleaned using a Dremel tool to remove matrix sediments. Samples were then inspected for sparry infill and, if present, only micrites were drilled to collect powder for isotopic analyses. The internal structure of calcite nodules, revealed by Scanning electron microscope imaging (Fig. S7), shows that calcite mainly occurs as secondary µm-scale, pore-filling cements associated with detrital quartz and clay minerals, suggesting pedogenic origin. Since the Red Clay formation has not been deeply buried, it is unlikely that the isotopic signals of these pedogenic carbonates were altered by burial diagenesis, consistent with the reasonable surface temperature recovered by clumped isotope analysis. Moreover, the coherent time-series variations in the stable isotope compositions of pedogenic carbonates across different sections, observed in both this study and previous work^6,60^, further support the preservation of pristine isotopic signals.

### Stable oxygen isotope analysis

Sample splits of approximately 200 μg were loaded into exetainers and He-flushed before analysis. δ^18^O analyses were carried out separately in two different labs. For Shilou and Jiaxian samples, sample powders were acidified with 103% phosphoric acid for 2–12 h at 50 °C. The stable isotopic compositions of released CO_2_ were measured using a Thermo Gasbench II coupled to a continuous flow Thermo 253 isotope ratio mass spectrometer (IRMS) at the University of Texas at Austin. The isotopic values are reported in per mil (‰) notation relative to Vienna Pee Dee Belemnite standard (VPDB) and were normalized to that scale using an in-house laboratory standard (UT Marble), NBS-18, and NBS-19. The external reproducibility of replicate analyses of standards averaged ±0.07‰ for δ^18^O (1σ). For Lantian samples, the measurements were performed on a Finnigan DELTAplusXP IRMS attached to a Thermo GasBench II (75 °C reaction with 100% phosphoric acid) at Nanjing University. The isotopic values were adjusted to VPDB scale using NBS-18, NBS-19, and an in-house laboratory standard (TTB-1). The standard error (1σ) of replicate analyses of the standard is ±0.04‰. To ensure measurements from different labs are comparable, four samples were measured for bulk isotopic composition in both labs, and the δ^18^O differences are within 0.1‰.

### Clumped isotope analysis

Clumped isotope measurements were carried out at the University of Washington Isolab, using two different instruments. The Jiaxian samples (*n* = 18) were analyzed on a Thermo MAT 253 IRMS configured to measure m/z 44-49 inclusive, following method described in Burgener et al. (2016)^61^ and Schauer et al. (2016)^62^. For each replicate analysis, sample aliquots containing 6–8 mg of CaCO_3_ were reacted in a common phosphoric acid bath at 90 °C for 10 min. The CO_2_ evolved from the acid digestion was purified cryogenically on an automated stainless steel/nickel vacuum line using a liquid N_2_ trap, an ethanol-dry ice mixture (~ −80 °C), and a Porapak Q trap (50/80 mesh, 122 cm long, 6.35 cm OD) at −20 °C. The CO_2_ was transferred through the Porapak using He carrier gas, isolated cryogenically, and flame-sealed into a Pyrex break seal before analysis. CO_2_ was introduced into the IRMS using an automated 10-port tube cracker inlet system. Isotope ratios were converted to the VPDB scale using intra and interlaboratory standards calibrated against international standards NBS-18 and NBS-19. These include in-house calcites with dissimilar bulk composition (C64 and C2, which are both reagent grade; and a *Porites* coral sample) and ETH standards (ETH 1–4). For each four carbonate sample unknowns, a carbonate standard was digested, purified, and analyzed using the same procedure. All sample unknowns were analyzed in triplicate at minimum.

The Lantian and Shilou samples (*n* = 14) were analyzed using a fully automated Nu Instruments NuCarb carbonate device coupled to a Nu Perspective IRMS. Sample powders containing approximately 1.2–1.5 mg calcite were loaded in individual glass vials before reaction with phosphoric acid at 70 °C, purification using cryogenic and Porapak traps, and analysis. For each of the five carbonate sample unknowns, a carbonate standard was measured, including eight intra and interlaboratory standards (IAEA-C1, IAEA-C2, MERCK, Coral, ETH 1–4). All sample unknowns were analyzed in triplicate at minimum.

Δ_47_ values were calculated using established methods^63^, with the exception that we used updated ^17^O correction parameters^64^, following recommendations of ref. ^65^ and ref. ^62^. Samples analyzed using the Thermo MAT 253 IRMS were corrected to CDES-90 °C acid values using CO_2_ equilibrated at 4, 60, and 1000 °C. Δ_47_ uncertainties for individual samples were given as one standard error (1σ) of replicate analyses, calculated using the long-term standard deviation of carbonate standard analyses, or sample replicates, whichever is larger. Samples analyzed using the fully automated NuCarb-Nu Perspective system were standardized to I-CDES^66^ 90 °C acid values using ETH1–4, IAEA-C1, IAEA-C2, and MERCK standards^67^, and calculations following D47Crunch pooled standardization with fully propagated Δ_47_ uncertainties^68^. The I-CDES and CDES scales are mathematically identical and interchangeable^67^, confirmed by agreement of ETH standard values from the MAT 253-CDES dataset with the accepted values^67^ used to anchor the Nu-system data to the I-CDES scale (see Supplementary Tables). Δ_47_ uncertainties for individual samples were given as one standard error (1σ) of replicate analyses, calculated using the long-term standard deviation of carbonate standard analyses, or sample replicates, whichever is larger. Clumped isotope temperature (T_Δ47_) values reported here were calculated from mean Δ_47_ values using the ref. ^69^ calibration for samples digested at 90 °C (no acid fractionation correction).

### Triple oxygen isotope analysis

Triple oxygen isotope measurements were carried out at the University of New Mexico Center for Stable Isotopes using two different instruments. The Jiaxian samples (*n* = 8) were measured on a Thermo Fisher MAT 253^+^ IRMS configured for triple oxygen isotope analyses. Sample powders containing at least 300 μg CaCO_3_ were first converted to CO_2_ using the phosphoric acid digestion system (25 °C reaction with 100% phosphoric acid for 16 h). The resulting gas was purified using a cryogenic trap to remove water and any non-condensable gases. CO_2_ was then fluorinated to produce O_2_ to be measured for triple oxygen isotopes using the nickel bomb fluorination method modified by ref. ^70^. No replicate analysis was carried out.

Lantian and Shilou samples (*n* = 14) were analyzed using a Tunable Infrared Laser Direct Absorption Spectroscopy (TILDAS) isotope analyzer designed and manufactured by Aerodyne Research, Inc. (ARI, Billerica, MA, USA), configured for triple oxygen isotope analyses of CO_2_. Detailed descriptions of the TILDAS and measurement procedure are provided in ref. ^71^. Before the analyses, sample aliquots containing 0.5 mg CaCO_3_ were loaded in exetainer vials sealed with a rubber septum. The vials were placed in a 70 °C heating block and He-flushed with a flow rate of 11 mL/min for 2 h. 5 ml of phosphoric acid was then injected into the vial and reacted with the sample powder for 8 h. The evolved CO_2_ was cryogenically separated from water and purified before being introduced into the TILDAS analyzer. For analysis in the TILDAS system, CO_2_ needs to be thoroughly mixed with CO_2_-free dry air in a two-bellow system, which produces a mixture of 456 ppm CO_2_. The isotope values of each sample measurement were determined relative to the reference gas by interpolating the prior and subsequent reference gas measurements in time. The total time required for 12 reference-sample cycles was 30 min. To monitor long-term analytical error and to place carbonate Δ^‘17^O values on the VPDB scale, interlaboratory standards (NBS-19, IAEA 603) were measured after four sample unknowns. The external reproducibility of replicate analyses of standards averaged 5 per meg for Δ^‘17^O (1σ). All sample unknowns were analyzed in triplicate at minimum.

### Calculating the δ^18^O and Δ^‘17^O of soil waters

With T_Δ47_ (°C), δ^18^O_c_ (‰, VSMOW), and δ^17^O_c_ (‰, VSMOW) available, δ^18^O_sw_ and Δ^*‘*17^O_sw_ can be calculated using the temperature-dependent fractionation factors between calcite and water (${}^{1{{{\rm{x}}}}/16}\alpha _{{{{\rm{calcite}}}}-{{{\rm{water}}}}}$)^72,73^:

1 $${}^{18/16}\alpha _{{{{\rm{calcite}}}}-{{{\rm{water}}}}}=\exp ((16.1\times 1000/(273.15+{{{{\rm{T}}}}}_{\varDelta 47})-24.6)/1000)$$

2 $${}^{17/16}\alpha _{{{{\rm{calcite}}}}-{{{\rm{water}}}}}={({}^{18/16}\alpha _{{{{\rm{calcite}}}}-{{{\rm{water}}}}})}^{\theta }$$

3 $${{{\rm{\theta }}}}=-1.39/(273.15+{{{{\rm{T}}}}}_{\Delta 47})+0.5305$$

The δ^18^O_sw_ (‰, VSMOW) and Δ^*‘*17^O_sw_ (per meg, VSMOW) can then be calculated using the following equations:

4 $${\delta }^{1{{{\rm{x}}}}}{{{{\rm{O}}}}}_{{{{\rm{sw}}}}}=({\delta }^{1{{{\rm{x}}}}}{{{{\rm{O}}}}}_{{{{\rm{c}}}}}+1000)/{}^{1{{{\rm{x}}}}/16}\alpha _{{{{\rm{calcite}}}}-{{{\rm{water}}}}}-1000$$

5 $${{{{\rm{\delta }}}}}^{{\prime} 1{{{\rm{x}}}}}{{{{\rm{O}}}}}_{{{{\rm{sw}}}}}=1000\times {{\mathrm{ln}}}({{{{\rm{\delta }}}}}^{1{{{\rm{x}}}}}{{{{\rm{O}}}}}_{{{{\rm{c}}}}}/1000+1)$$

6 $${\Delta }^{{\prime} 17}{{{{\rm{O}}}}}_{{{{\rm{sw}}}}}=1000\times ({{{{\rm{\delta }}}}}^{{\prime} 17}{{{{\rm{O}}}}}_{{{{\rm{sw}}}}}-0.528\times {\,{{{\rm{\delta }}}}}^{{\prime} 18}{{{{\rm{O}}}}}_{{{{\rm{sw}}}}})$$

Where the superscript 1x refers to either 18 or 17.

### Reconstructing rainfall δ^18^O

The δ^18^O of pedogenic carbonates (δ^18^O_c_) record soil-water δ^18^O (δ^18^O_sw_), with the formation temperature constrained by clumped isotopes (T_Δ47_, see “Methods”). In well-drained soils, δ^18^O_sw_ inherits the δ^18^O of local rainfall (δ^18^O_p_), which is controlled by the regional water cycle and large-scale air circulation^21^. However, δ^18^O_sw_ values are also affected by soil processes such as evaporation and mixing^74^. The triple oxygen isotope (^16^O-^17^O-^18^O) compositions of pedogenic carbonates can help quantify the degree of evaporation experienced by soil waters from which the carbonates precipitated, thereby improving estimates of δ^18^O_p_^19^. Δ′^17^O represents the deviation in δ′^17^O-δ′^18^O space (δ′ = 1000 × ln(δ/1000 + 1)) from the global meteoric water line (GMWL, Δ′^17^O = δ′^17^O-λ × δ′^18^O, λ = 0.528)^75^. Unlike δ^18^O_p_, Δ′^17^O_p_ is not sensitive to processes involving equilibrium fractionation such as condensation and rainout from air masses that control the GMWL^75^. Consequently, Δ′^17^O_sw_ values are primarily governed by evaporation-induced kinetic fractionation, which has a trajectory in δ′^17^O-δ′^18^O space shallower than the GMWL slope^19^. Analyses of modern waters and soil waters reconstructed from a global compilation of Holocene pedogenic carbonates support the use of Δ′^17^O as a proxy of evaporation and thus regional aridity, with higher evaporation corresponding to lower Δ′^17^O_sw_ and δ^18^O_sw_^19,76^. Here we reconstructed δ^18^O_sw_ and Δ′^17^O_sw_ using δ^18^O, T_Δ47_, and Δ′^17^O of calcite nodules (Fig. S2). By leveraging Δ′^17^O_sw_, we accounted for evaporation and reconstructed δ^18^O_p_.

### Bayesian inversion

To quantitatively constrain environmental variables based on proxy data, here we inverted for δ^18^O_p_, MAST, MAP, soil water evaporation rate, and RH, with a quasi-steady-state soil water isotope model^19^ using a Markov Chain Monte Carlo approach conditioned on the proxy data (δ^18^O_c_, Δ′^17^O_c_, Δ_47_)^77^. The steady-state model quantifies the effect of evaporation on δ^18^O_sw_ and Δ′^17^O_sw_ as a function of soil depth, RH, MAST, and evaporation rate. The analyses were performed in R version 4.4.2^78^ using the rjags^79^ and R2jags package^80^. To avoid biased interpretation of our proxy data, we set loose prior ranges for all the related environmental inputs, including RH (10–90%,), δ^18^O_p_ (−25 to −5‰), evaporation rate (10^−10^–10^−9^ m/s), MAP (normal distribution, mean = 500 mm, SD = 100 mm), and MAST (normal distribution, mean = 10, SD = 5 °C), based on either theoretical constraints^19^ or modern observations across the CLP. These weak prior constraints allowed the model to fully explore environmental parameter space and conservatively determine that δ^18^O_p_ is likely the predominant control on δ^18^O_sw_. We ran three parallel chains, each consisting of 5 × 10^5^ iterations, with a burn-in of 1 × 10^5^. Convergence was assessed by the Gelman and Rubin convergence factor (Rhat) and effective sample sizes reported by rjags^81^. All the posteriors show strong convergence (Rhat <1.03).

### CESM2 experiments

The climate model simulations analyzed in this study are run with the CESM2^82^. CESM2 incorporates Community Atmospheric Model version 6, Community Land Model version 5, Community Ice Code version 5, and Parallel Ocean Program version 2. The atmosphere and land components are run at a 0.9° × 1.25° horizontal resolution, while the ocean and sea ice components are run with a nominal 1° resolution. The pre-industrial simulation included in this study is part of the Climate Model Intercomparison Project phase 6 Tier 1 experiment (*piControl.001*)^82^.

The mid-Pliocene simulations (*midPliocene-eoi400*) analyzed in this study have been reported in ref. ^34^, targeted at the mid-Piacenzian Warm Period (MPWP, 3.205 Ma). The MPWP boundary conditions follow the protocol of the second phase of the PlioMIP (PlioMIP2)^33^, including present-day orbital configuration, continental greening, a reduced Greenland Ice Sheet, a deglaciated western Antarctica, and adjusted topography and coastlines^34,83^. Notably, the Bering and Canadian Arctic Archipelago straits are closed, and the Sunda, Sahul, Baltic Shelves, and Hudson Bay are exposed.

In addition, to assess the climate changes induced by changes in CO_2_ during the Pliocene, we report a new fully coupled mid-Pliocene simulation where the same MPWP protocol is used except that the CO_2_ concentration is reduced to the pre-industrial level at 280 ppm (*midPliocene-eoi280)*.

### Recalculating SST records

To further investigate the evolution of the MTG, we collated high-quality ${{\mbox{U}}}_{37}^{k\hbox{'}}$-based SST records around the global ocean^27^. These SST records were divided into three groups based on their latitudes: high-latitude >50°N regions (ODP 883/884, 907, 982), mid-latitude regions (20–50°N: ODP 1010, 1021, and 1208), and tropics (<20°N: ODP 722, 846, 850, U1338, 1241) (Fig. S1). To account for the seasonality of alkenones and nonlinear response of ${{\mbox{U}}}_{37}^{k\hbox{'}}$ to temperature, we recalculated the ${{\mbox{U}}}_{37}^{k\hbox{'}}$-based SSTs by applying the original ${{\mbox{U}}}_{37}^{k\hbox{'}}$ data to a Bayesian B-spline regression model, BAYSPLINE^84^. The integrated SSTs of each group were computed by binning the SSTs from each ODP site to 100-kyr windows and averaging the estimates. The SST differences between the tropics and mid-latitude regions and the tropics and high-latitude regions were used to infer the MTG.

## Supplementary information

Supplementary Information

Transparent Peer Review file

## Data Availability

The data generated in this study, including measured δ^18^O, Δ_47_, and Δ^’17^O data of pedogenic carbonates, have been deposited in the Figshare database^85^.

## Code availability

Codes and the associated data used for modeling, statistical analyses, and plotting figures have been deposited in Zenodo^86^.

## References

[1] Steinthorsdottir, M. et al. The Miocene: the future of the past. *Paleoceanogr. Paleoclimatol.* **36**, e2020PA004037 (2021).

[2] Peppe, D. J. et al. Oldest evidence of abundant C4 grasses and habitat heterogeneity in eastern Africa. *Science* **380**, 173–177 (2023). 37053309 10.1126/science.abq2834

[3] Quade, J., Cerling, T. E. & Bowman, J. R. Development of Asian monsoon revealed by marked ecological shift during the latest Miocene in northern Pakistan. *Nature* **342**, 163 (1989).

[4] Andrae, J. W. et al. Initial expansion of C4 vegetation in Australia during the late Pliocene. *Geophys. Res. Lett.* **45**, 4831–4840 (2018).

[5] The Cenozoic CO_2_ Proxy Integration Project (CENCO2PIP) Consortium. Toward a Cenozoic history of atmospheric CO_2_. *Science* **382**, eadi5177 (2023). 10.1126/science.adi5177 38060645

[6] Passey, B. H. et al. Strengthened East Asian summer monsoons during a period of high-latitude warmth? Isotopic evidence from Mio-Pliocene fossil mammals and soil carbonates from northern China. *Earth Planet. Sci. Lett.* **277**, 443–452 (2009).

[7] Edwards, E. J., Osborne, C. P., Strömberg, C. A. & Smith, S. A. C4 Grasses Consortium. The origins of C4 grasslands: integrating evolutionary and ecosystem science. *Science* **328**, 587–591 (2010). 20431008 10.1126/science.1177216

[8] Da, J., Zhang, Y. G., Li, G. & Ji, J. Aridity-driven decoupling of δ13C between pedogenic carbonate and soil organic matter. *Geology*10.1130/G47241.1 (2020).

[9] Lu, J. et al. The Early Pliocene global expansion of C4 grasslands: a new organic carbon-isotopic dataset from the north China plain. *Palaeogeogr. Palaeoclimatol. Palaeoecol.*10.1016/j.palaeo.2019.109454 (2019).

[10] Zhou, B. et al. Late Pliocene–Pleistocene expansion of C4 vegetation in semiarid East Asia linked to increased burning. *Geology* **42**, 1067–1070 (2014).

[11] Shen, X. et al. Increased seasonality and aridity drove the C4 plant expansion in Central Asia since the Miocene–Pliocene boundary. *Earth Planet. Sci. Lett.* **502**, 74–83 (2018).

[12] Zhu, Z. et al. Hominin occupation of the Chinese Loess Plateau since about 2.1 million years ago. *Nature* **559**, 608–612 (2018). 29995848 10.1038/s41586-018-0299-4

[13] Zhou, B. et al. Hominin response to oscillations in climate and local environments during the mid-pleistocene climate transition in Northern China. *Geophys. Res. Lett.* **50**, e2023GL104931 (2023).

[14] Cerling, T. E. et al. Woody cover and hominin environments in the past 6 million years. *Nature* **476**, 51–56 (2011). 21814275 10.1038/nature10306

[15] Chiang, J. C. H. et al. Role of seasonal transitions and Westerly Jets in East Asian paleoclimate. *Quat. Sci. Rev.* **108**, 111–129 (2015).

[16] Ding, Y. & Chan, J. C. L. The East Asian summer monsoon: an overview. *Meteorol. Atmos. Phys.* **89**, 117–142 (2005).

[17] Zhang, H. et al. East Asian hydroclimate modulated by the position of the westerlies during Termination I. *Science* **362**, 580–583 (2018). 30385577 10.1126/science.aat9393

[18] Tian, C., Wang, L., Tian, F., Zhao, S. & Jiao, W. Spatial and temporal variations of tap water 17O-excess in China. *Geochim. Cosmochim. Acta* **260**, 1–14 (2019).

[19] Kelson, J. R. et al. Triple oxygen isotope compositions of globally distributed soil carbonates record widespread evaporation of soil waters. *Geochim. Cosmochim. Acta*10.1016/j.gca.2023.06.034 (2023).

[20] Cheng, H. et al. Ice age terminations. *Science* **326**, 248–252 (2009). 19815769 10.1126/science.1177840

[21] Chiang, J. C. H., Herman, M. J., Yoshimura, K. & Fung, I. Y. Enriched East Asian oxygen isotope of precipitation indicates reduced summer seasonality in regional climate and westerlies. *Proc. Natl. Acad. Sci. USA* **117**, 14745 (2020). 32532921 10.1073/pnas.1922602117 PMC7334498

[22] Abell, J. T., Winckler, G., Anderson, R. F. & Herbert, T. D. Poleward and weakened westerlies during Pliocene warmth. *Nature* **589**, 70–75 (2021). 33408375 10.1038/s41586-020-03062-1

[23] Son, J.-H., Seo, K.-H. & Wang, B. How does the tibetan plateau dynamically affect downstream monsoon precipitation? *Geophys. Res. Lett.* **47**, e2020GL090543 (2020).

[24] He, L. et al. Northward extension of east asian summer monsoon since the Miocene set by the uplift of Tibetan Plateau. *Geophys. Res. Lett.* **51**, e2023GL107262 (2024).

[25] Rea, D. K., Snoeckx, H. & Joseph, L. H. Late Cenozoic eolian deposition in the north Pacific: Asian drying, Tibetan uplift, and cooling of the Northern hemisphere. *Paleoceanography* **13**, 215–224 (1998).

[26] Zhong, Y. et al. Humidification of Central Asia and equatorward shifts of westerly winds since the late Pliocene. *Commun. Earth Environ.* **3**, 1–9 (2022).

[27] Herbert, T. D. et al. Late Miocene global cooling and the rise of modern ecosystems. *Nat. Geosci.* **9**, 843–847 (2016).

[28] Lu, H. et al. Decoupled land and ocean temperature trends in the early-middle Pleistocene. *Geophys. Res. Lett.* **49**, e2022GL099520 (2022).

[29] Sun, Y., An, Z., Clemens, S. C., Bloemendal, J. & Vandenberghe, J. Seven million years of wind and precipitation variability on the Chinese Loess Plateau. *Earth Planet. Sci. Lett.* **297**, 525–535 (2010).

[30] Kelson, J. R. et al. A proxy for all seasons? A synthesis of clumped isotope data from Holocene soil carbonates. *Quat. Sci. Rev.* **234**, 106259 (2020).

[31] Da, J., Li, G. K. & Ji, J. Seasonal changes in the formation time of pedogenic carbonates on the Chinese Loess Plateau during Quaternary glacial cycles. *Quat. Sci. Rev.* **305**, 108008 (2023).

[32] Breecker, D. O., Sharp, Z. D. & McFadden, L. D. Seasonal bias in the formation and stable isotopic composition of pedogenic carbonate in modern soils from central New Mexico, USA. *Geol. Soc. Am. Bull.* **121**, 630–640 (2009).

[33] Haywood, A. et al. The Pliocene model intercomparison project (PlioMIP) phase 2: scientific objectives and experimental design. *Climate* **12**, 663–675 (2016).

[34] Feng, R., Otto-Bliesner, B. L., Brady, E. C. & Rosenbloom, N. Increased climate response and earth system sensitivity from CCSM4 to CESM2 in mid-pliocene simulations. *J. Adv. Model. Earth Syst.* **12**, e2019MS002033 (2020).

[35] He, L., Zhou, T., Chen, X., Zuo, M. & Zou, L. *Earlier Seasonal March of the East Asian Summer Monsoon in the Mid-Pliocene*. 10.1175/JCLI-D-23-0709.1 (2024).

[36] Ao, H. et al. Late Miocene–Pliocene Asian monsoon intensification linked to Antarctic ice-sheet growth. *Earth Planet. Sci. Lett.* **444**, 75–87 (2016).

[37] Li, F., Rousseau, D.-D., Wu, N., Hao, Q. & Pei, Y. Late Neogene evolution of the East Asian monsoon revealed by terrestrial mollusk record in Western Chinese Loess Plateau: from winter to summer dominated sub-regime. *Earth Planet. Sci. Lett.* **274**, 439–447 (2008).

[38] Zheng, Y. et al. Severe drought conditions in Northern East Asia during the early pliocene caused by weakened Pacific meridional temperature gradient. *Geophys. Res. Lett.* **49**, e2022GL098813 (2022).

[39] Wang, X., Nie, J., Stevens, T., Zhang, H. & Xiao, W. Resolving conflicting models of late Miocene East Asian summer monsoon intensity recorded in Red Clay deposits on the Chinese Loess Plateau. *Earth-Sci. Rev.*10.1016/j.earscirev.2022.104200 (2022).

[40] Ahmed, I. A. M. & Maher, B. A. Identification and paleoclimatic significance of magnetite nanoparticles in soils. *Proc. Natl. Acad. Sci. USA*. **115**, 1736–1741 (2018). 29432151 10.1073/pnas.1719186115 PMC5828620

[41] Fan, J., Jones, S. B., Qi, L. B., Wang, Q. J. & Huang, M. B. Effects of precipitation pulses on water and carbon dioxide fluxes in two semiarid ecosystems: measurement and modeling. *Environ. Earth Sci.* **67**, 2315–2324 (2012).

[42] Cowie, R. H. The life-cycle and productivity of the land snail Theba pisana (Mollusca: Helicidae). *J. Animal Ecol.* **53**, 311–325 (1984).

[43] Deng, L., Jia, G., Jin, C. & Li, S. Warm season bias of branched GDGT temperature estimates causes underestimation of altitudinal lapse rate. *Org. Geochem.* **96**, 11–17 (2016).

[44] Wang, H. et al. New calibration of terrestrial brGDGT paleothermometer deconvolves distinct temperature responses of two isomer sets. *Earth Planet. Sci. Lett.* **626**, 118497 (2024).

[45] Clemens, S. C., Prell, W. L., Sun, Y., Liu, Z. & Chen, G. Southern Hemisphere forcing of Pliocene δ18O and the evolution of Indo-Asian monsoons. *Paleoceanography* **23**, PA4210 (2008).

[46] Wen, Y. et al. CO2-forced Late Miocene cooling and ecosystem reorganizations in East Asia. *Proc. Natl. Acad. Sci. USA*. **120**, e2214655120 (2023). 36689658 10.1073/pnas.2214655120 PMC9945954

[47] An, Z. et al. Multiple expansions of C4 plant biomass in East Asia since 7 Ma coupled with strengthened monsoon circulation. *Geology* **33**, 705–708 (2005).

[48] Sun, J., Lü, T., Zhang, Z., Wang, X. & Liu, W. Stepwise expansions of C4 biomass and enhanced seasonal precipitation and regional aridity during the Quaternary on the southern Chinese Loess Plateau. *Quat. Sci. Rev.* **34**, 57–65 (2012).

[49] Long, H. et al. The westerlies-monsoon interaction shaped asymmetric lake expansions over the Tibetan Plateau in warming periods. *Sci. Bull.*10.1016/j.scib.2025.07.023 (2025). 10.1016/j.scib.2025.07.023 40816960

[50] Kong, W., Swenson, L. M. & Chiang, J. C. H. Seasonal transitions and the westerly jet in the Holocene East Asian summer monsoon. *J. Clim.* **30**, 3343–3365 (2017).

[51] Huybers, P. & Wunsch, C. Obliquity pacing of the late Pleistocene glacial terminations. *Nature* **434**, 491–494 (2005). 15791252 10.1038/nature03401

[52] Li, M., Hinnov, L. A., Huang, C. & Ogg, J. G. Sedimentary noise and sea levels linked to land–ocean water exchange and obliquity forcing. *Nat. Commun.* **9**, 1004 (2018). 29520064 10.1038/s41467-018-03454-y PMC5843644

[53] Qin, J. et al. 1.2 Myr band of earth-mars obliquity modulation on the evolution of cold late miocene to warm early pliocene climate. *J. Geophys. Res.: Solid Earth* **127**, e2022JB024131 (2022).

[54] Retallack, G. J. Pedogenic carbonate proxies for amount and seasonality of precipitation in paleosols. *Geology* **33**, 333–336 (2005).

[55] An, Z. et al. Late Cenozoic climate change in monsoon-arid Asia and global changes. In An, Z., *Late Cenozoic Climate Change in Asia* 491–581 (Springer, 2014).

[56] Liu, X. et al. Magnetic properties of the Tertiary red clay from Gansu. *Sci. China Ser. D.-Earth Sci.* **44**, 635–651 (2001).

[57] Sun, D., Liu, D., Chen, M., An, Z. & John, S. Magnetostratigraphy and palaeoclimate of Red Clay sequences from Chinese Loess Plateau. *Sci. China Ser. D.-Earth Sci.* **40**, 337–343 (1997).

[58] Qiang, X. K., Li, Z.-X., Powell, C. M. & Zheng, H. B. Magnetostratigraphic record of the Late Miocene onset of the East Asian monsoon, and Pliocene uplift of northern Tibet. *Earth Planet. Sci. Lett.* **187**, 83–93 (2001).

[59] Retallack, G. J. Refining a pedogenic-carbonate CO_2_ paleobarometer to quantify a middle Miocene greenhouse spike. *Palaeogeogr., Palaeoclimatol., Palaeoecol.* **281**, 57–65 (2009).

[60] Gallagher, T. M. et al. Regional patterns in Miocene-Pliocene aridity across the Chinese Loess Plateau revealed by high resolution records of paleosol carbonate and occluded organic matter. *Paleoceanogr. Paleoclimatol.* **36**, e2021PA004344 (2021).

[61] Burgener, L. et al. Variations in soil carbonate formation and seasonal bias over >4 km of relief in the western Andes (30°S) revealed by clumped isotope thermometry. *Earth Planet. Sci. Lett.* **441**, 188–199 (2016).

[62] Schauer, A. J., Kelson, J., Saenger, C. & Huntington, K. W. Choice of 17O correction affects clumped isotope (Δ47) values of CO2 measured with mass spectrometry. *Rapid Commun. Mass Spectrom.* **30**, 2607–2616 (2016). 27650267 10.1002/rcm.7743

[63] Huntington, K. W. et al. Methods and limitations of ‘clumped’ CO2 isotope (Δ47) analysis by gas-source isotope ratio mass spectrometry. *J. Mass Spectrom.* **44**, 1318–1329 (2009). 19621330 10.1002/jms.1614

[64] Brand, W. A., Assonov, S. S. & Coplen, T. B. Correction for the 17O interference in δ(13C) measurements when analyzing CO2 with stable isotope mass spectrometry (IUPAC Technical Report). *Pure Appl. Chem.* **82**, 1719–1733 (2010).

[65] Daëron, M., Blamart, D., Peral, M. & Affek, H. P. Absolute isotopic abundance ratios and the accuracy of Δ47 measurements. *Chem. Geol.* **442**, 83–96 (2016).

[66] Dennis, K. J., Affek, H. P., Passey, B. H., Schrag, D. P. & Eiler, J. M. Defining an absolute reference frame for ‘clumped’ isotope studies of CO_2_. *Geochim. Cosmochim. Acta* **75**, 7117–7131 (2011).

[67] Bernasconi, S. M. et al. InterCarb: a community effort to improve interlaboratory standardization of the carbonate clumped isotope thermometer using carbonate standards. *Geochem. Geophys. Geosyst.* **22**, e2020GC009588 (2021). 34220359 10.1029/2020GC009588 PMC8244079

[68] Daëron, M. Full propagation of analytical uncertainties in Δ47 measurements. *Geochem. Geophys. Geosyst.* **22**, e2020GC009592 (2021).

[69] Anderson, N. T. et al. A unified clumped isotope thermometer calibration (0.5–1,100 °C) using carbonate-based standardization. *Geophys. Res. Lett.* **48**, e2020GL092069 (2021).

[70] Wostbrock, J. A. G., Cano, E. J. & Sharp, Z. D. An internally consistent triple oxygen isotope calibration of standards for silicates, carbonates and air relative to VSMOW2 and SLAP2. *Chem. Geol.* **533**, 119432 (2020).

[71] Perdue, N., Sharp, Z., Nelson, D., Wehr, R. & Dyroff, C. A rapid high-precision analytical method for triple oxygen isotope analysis of CO_2_ gas using tunable infrared laser direct absorption spectroscopy. *Rapid Commun. Mass Spectrom.* **36**, e9391 (2022). 36056818 10.1002/rcm.9391 PMC9541814

[72] Tremaine, D. M., Froelich, P. N. & Wang, Y. Speleothem calcite farmed in situ: modern calibration of δ18O and δ13C paleoclimate proxies in a continuously-monitored natural cave system. *Geochim. Cosmochim. Acta* **75**, 4929–4950 (2011).

[73] Wostbrock, J. A. G. et al. Calibration of carbonate-water triple oxygen isotope fractionation: seeing through diagenesis in ancient carbonates. *Geochim. Cosmochim. Acta* **288**, 369–388 (2020).

[74] Fischer-Femal, B. J. & Bowen, G. J. Coupled carbon and oxygen isotope model for pedogenic carbonates. *Geochim. Cosmochim. Acta* **294**, 126–144 (2021).

[75] Luz, B. & Barkan, E. Variations of 17O/16O and 18O/16O in meteoric waters. *Geochim. Cosmochim. Acta* **74**, 6276–6286 (2010).

[76] Aron, P. G. et al. Triple oxygen isotopes in the water cycle. *Chem. Geol.* **565**, 120026 (2021).

[77] Lunn, D., Jackson, C., Best, N., Thomas, A. & Spiegelhalter, D. *The BUGS Book: A Practical Introduction to Bayesian Analysis.*(CRC Press, 2013).

[78] R Core Team. *R: A Language and Environment for Statistical Computing*. (R Foundation for Statistical Computing, Vienna, Austria, 2024).

[79] Plummer, M. *rjags: Bayesian Graphical Models Using MCMC* https://CRAN.Rproject.org/package=rjags (R package version 4-6, 2016, 2018).

[80] Su, Y.-S., Yajima, M., & Su, M. *Package ‘r2jags’* http://CRAN.R-project.org/package=R2jags (R package version 0.03-0, 2015).

[81] Gelman, A. & Rubin, D. B. Inference from Iterative simulation using multiple sequences. *Stat. Sci.* **7**, 457–472 (1992).

[82] Danabasoglu, G. et al. The Community Earth System Model version 2 (CESM2). *J. Adv. Model. Earth Syst.* **12**, e2019MS001916 (2020).

[83] Dowsett, H. et al. The PRISM4 (mid-Piacenzian) paleoenvironmental reconstruction. *Climate* **12**, 1519–1538 (2016).

[84] Tierney, J. E. & Tingley, M. P. BAYSPLINE: a new calibration for the alkenone paleothermometer. *Paleoceanogr. Paleoclimatol.* **33**, 281–301 (2018).

[85] Da, J. Rainy spring delayed the late Neogene expansion of C4 biomass over East Asia. 10.6084/m9.figshare.29137106.v3 (2025)

[86] Da, J. da-jiawei/pliocene_westerly_jet_C4: East Asia jet-C4 coupling. *Zenodo*10.5281/zenodo.18940073 (2026).

[87] Luo, X. et al. Mapping the global distribution of C4 vegetation using observations and optimality theory. *Nat. Commun.* **15**, 1219 (2024). 38336770 10.1038/s41467-024-45606-3 PMC10858286

[88] Schiemann, R., Lüthi, D. & Schär, C. Seasonality and interannual variability of the westerly jet in the Tibetan Plateau region. 10.1175/2008JCLI2625.1 (2009).

[89] Wickham, H. *Ggplot2: Elegant Graphics for Data Analysis* (Springer-Verlag New York, 2016).

[90] Posit team. *RStudio: Integrated Development Environment for R* (Posit Software, PBC, 2023).

[91] Lisiecki, L. E. & Raymo, M. E. A Pliocene-pleistocene stack of 57 globally distributed benthic δ 18 O records. *Paleoceanography* **20**, 1–17 (2005).

[92] Yang, S. et al. A strengthened East Asian summer monsoon during Pliocene warmth: evidence from ‘red clay’ sediments at Pianguan, northern China. *J. Asian Earth Sci.* **155**, 124–133 (2018).

