# Supplementary material for: Jet-induced rainfall seasonality and C4 migration over East Asia
Source: Nat Commun. 2026 Jun 10;17:7392. doi: 10.1038/s41467-026-74312-5 (PMC13402663; doi:10.1038/s41467-026-74312-5)
Supplement: Supplementary file 1 — Supplementary Information [file 41467_2026_74312_MOESM1_ESM.pdf]

Supporting Information for
Jet-Induced Rainfall Seasonality and C_4 Migration over East Asia

Jiawei Da[1,2*]†, Chijun Sun[3], Lily Serach[1], Timothy Gallagher[4], Huayu Lu[5], Katharine Huntington[6], Ran Feng[7], Hanzhi Zhang[5], Hanlin Wang[8], Shunchuan Ji[9], Zachary Sharp[10], Junfeng Ji[2], Daniel Breecker[1]

Jiawei Da
Email: jiawei@psu.edu

This PDF file includes:

Figures S1 to S6

Figures

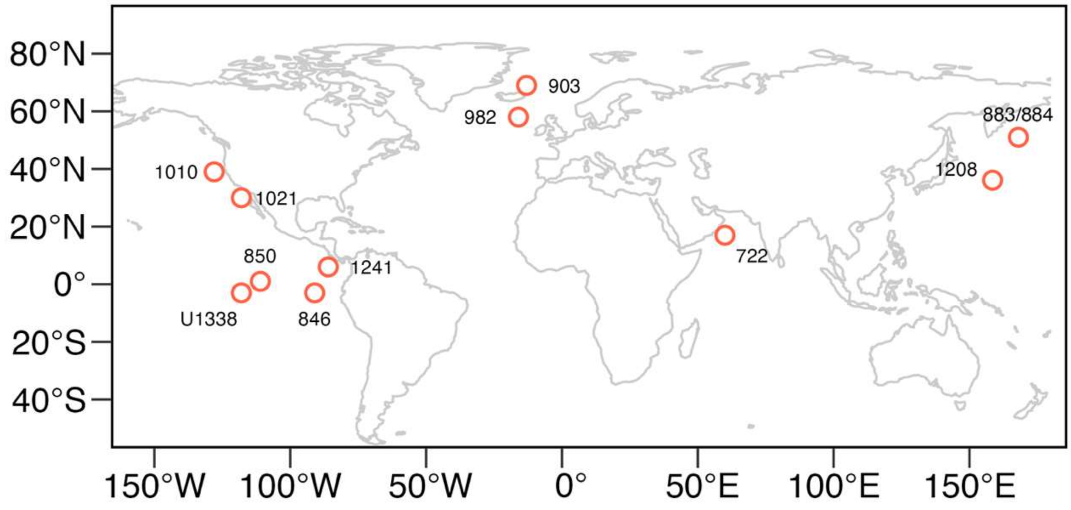

Fig. S1. **The locations of Ocean Drilling Program (ODP) sites used in this study [1].**

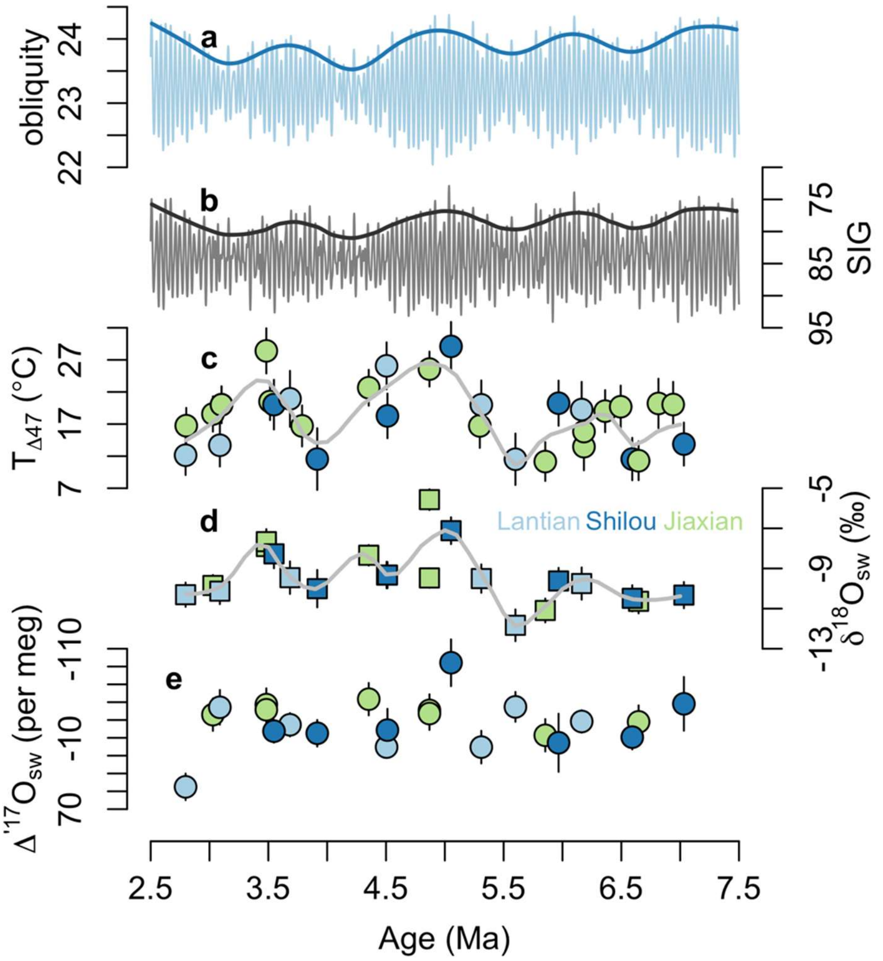

Fig. S2. Comparison of nodule-derived records with obliquity signal. (a) Obliquity signal in La2004 astronomical solution along with its filtered ~1.2-Myr amplitude modulation. (b) 25° - 65°N summer insolation gradient (SIG) with its filtered ~1.2-Myr amplitude modulation. (c) Clumped isotope temperature ($T_{\Delta47}$). Error bars denote standard errors from replicate analyses. (d) Reconstructed $\delta^{18}O$ of soil waters ($\delta^{18}O_{sw}$) using paired $\delta^{18}O_c$-$T_{\Delta47}$ measurements. Error bars are propagated from uncertainties in $\delta^{18}O_c$ and $T_{\Delta47}$ using Monte Carlo random sampling. (e) The $\Delta'^{17}O$ of soil waters ($\Delta'^{17}O_{sw}$) calculated from paired $T_{\Delta47}$-$\Delta'^{17}O_c$ measurements. Note the reversed scale for SIG and $\Delta'^{17}O_{sw}$. The grey lines in panels c-d are the LOESS lines (span = 0.3).

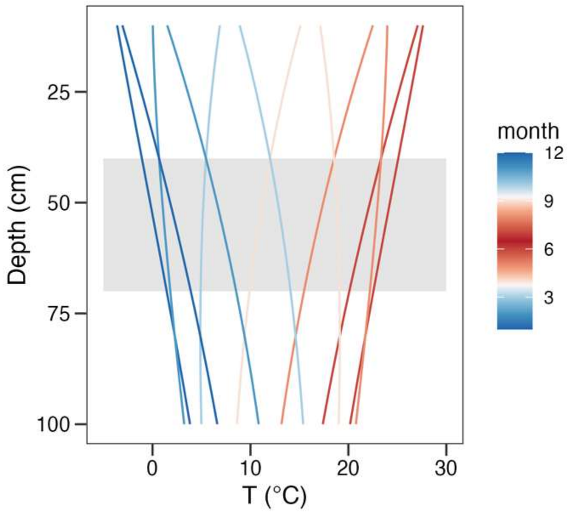

Fig. S3. Modeled depth profile of monthly soil temperature. Soil temperatures at various depths were simulated using a heat-diffusion model[2], with mean annual surface temperature (12°C) and the amplitude of temperature variation (17°C) based on mean values of modern observations across the CLP (data from https://www.webmap.cn/), and other soil parameters from Quade et al. (2013)[3].

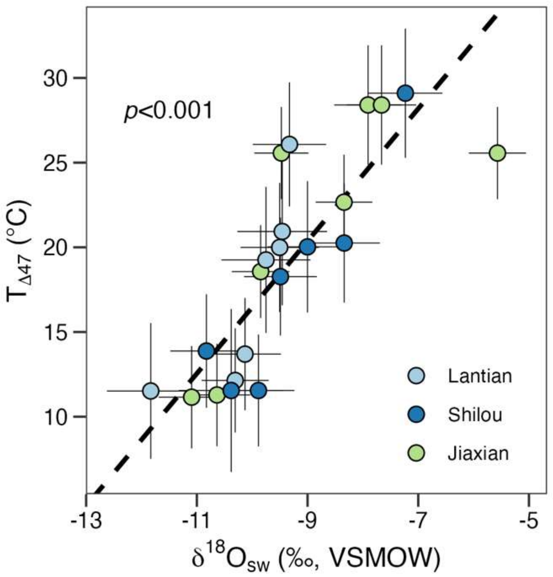

Fig. S4 Cross-plot of TΔ47 vs. δ18Osw, color-coded by different sections. The dashed line is the best fitting line based on York linear regression[4] that accounts for uncertainties associated with both parameters.

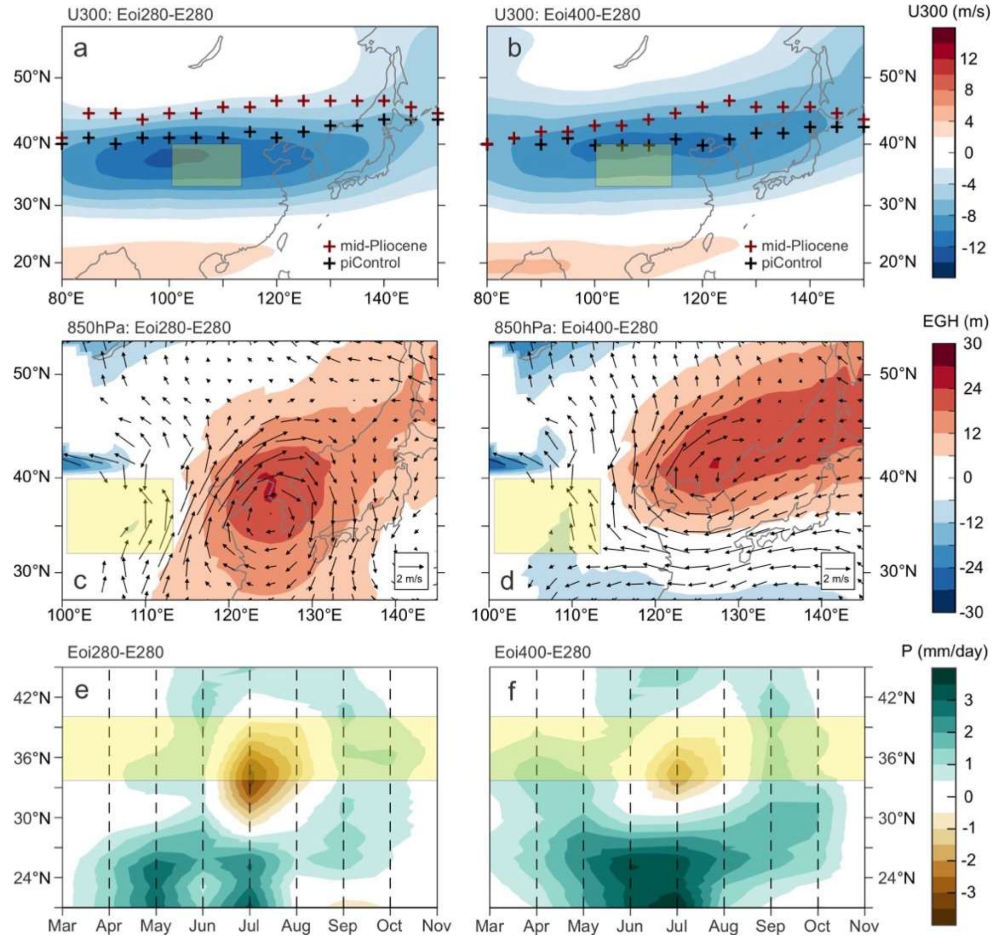

Fig. S5. The westerly jet, western Pacific subtropical high, and East Asian monthly rainfall from model simulations. (a-b) Differences in zonal winds at 300 hPa in July. The markers denote the axis of the westerly jet defined as the latitude with the maximum zonal wind speed over 10-50°N. (c-d) Differences in the eddy geopotential height (EGH, shading) and horizontal wind (vectors) at 850 hPa in July. (e-f) Monthly precipitation anomaly over 105-120°E. The left panels show the differences between Pliocene 280 ppm (Eoi280) and pre-industrial (E280) experiments, whereas the right panels show the differences between Pliocene 400 ppm (Eoi400) and E280 experiments. Yellow boxes highlight the CLP region.

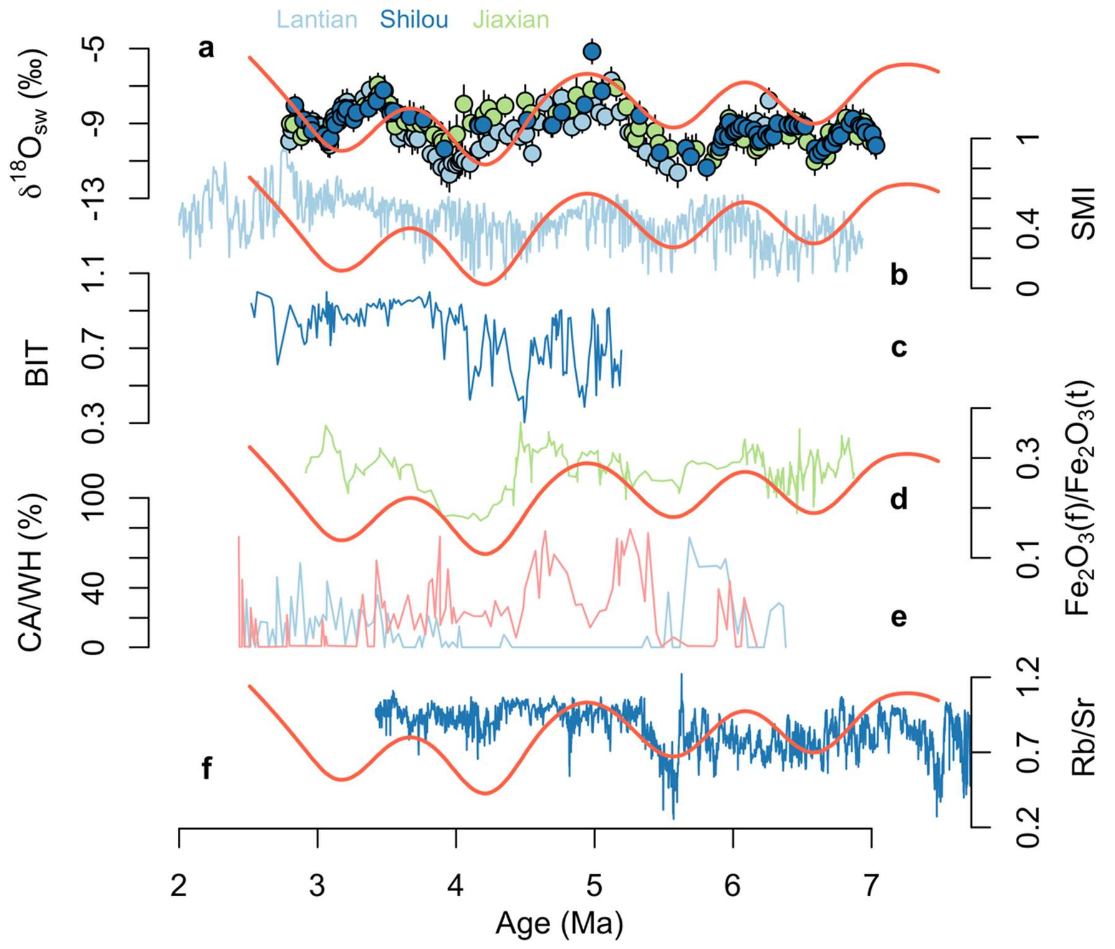

Fig. S6. Proxy-derived records from the CLP. (a) $\delta^{18}O_{sw}$ from this study. (b) Summer monsoon index based on soil magnetic susceptibility and carbonate content from Lingtai section[14]. (c) The brGDGT-based aridity indicators (BIT) from Shilou section [5]. (d) The ratio of free iron to total iron content from Pianguan section [6]. (e) Relative abundance of mollusk assemblages (red: warm-humid, blue: cold-dry) [7]. (f) Bulk soil Rb/Sr ratio from Shilou section [8]. Higher values in panels b-d and f were interpreted to indicate stronger monsoon and/or wetter climate. Red curves in panels a, b, and d show the ~1.2 Ma obliquity amplitude modulation.

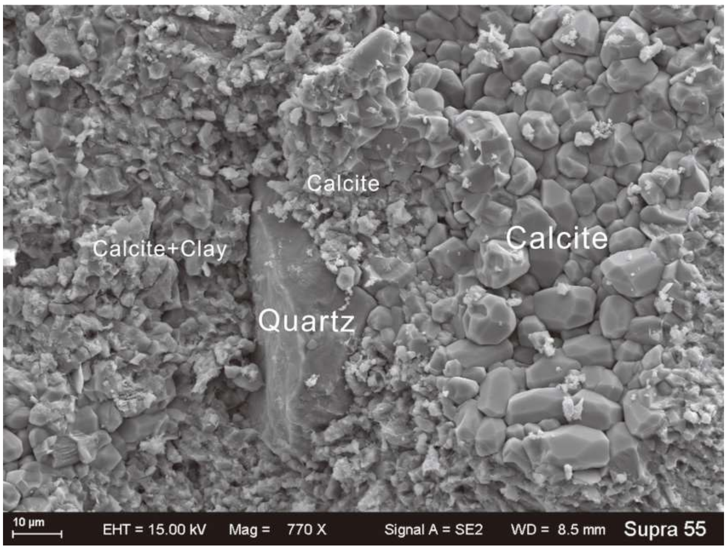

Figure S7 Scanning electron microscope (SEM) image of a calcite nodule from the Red Clay sequence at the Lantian section. Mineral phases shown in the image were confirmed by energy-dispersive X-ray spectroscopy (EDS).

Supplementary References

1. Herbert, T. D. *et al.* Late Miocene global cooling and the rise of modern ecosystems. *Nature Geoscience* **9**, (2016).

2. Hillel, D. *Fundamentals of Soil Physics*. (Academic press, 2013).

3. Quade, J., Eiler, J., Daeron, M. & Achyuthan, H. The clumped isotope geothermometer in soil and paleosol carbonate. *Geochimica Et Cosmochimica Acta* **105**, 92–107 (2013).

4. York, D., Evensen, N. M., Martínez, M. L. & De Basabe Delgado, J. Unified equations for the slope, intercept, and standard errors of the best straight line. *Am. J. Phys.* **72**, 367–375 (2004).

5. Zheng, Y. *et al.* Severe Drought Conditions in Northern East Asia During the Early Pliocene Caused by Weakened Pacific Meridional Temperature Gradient. *Geophysical Research Letters* **49**, e2022GL098813 (2022).

6. Yang, S. *et al.* A strengthened East Asian Summer Monsoon during Pliocene warmth: Evidence from 'red clay' sediments at Pianguan, northern China. *Journal of Asian Earth Sciences* **155**, 124–133 (2018).

7. Li, F., Rousseau, D.-D., Wu, N., Hao, Q. & Pei, Y. Late Neogene evolution of the East Asian monsoon revealed by terrestrial mollusk record in Western Chinese Loess Plateau: From winter to summer dominated sub-regime. *Earth and Planetary Science Letters* **274**, 439–447 (2008).

8. Ao, H. *et al.* Global warming-induced Asian hydrological climate transition across the Miocene–Pliocene boundary. *Nature Communications* **12**, 6935 (2021).
