# Supplementary material for: Jet-induced rainfall seasonality and C4 migration over East Asia
Source: Nat Commun. 2026 Jun 10;17:7392. doi: 10.1038/s41467-026-74312-5 (PMC13402663; doi:10.1038/s41467-026-74312-5)
Supplement: Supplementary file 2 — Transparent Peer Review file [file 41467_2026_74312_MOESM2_ESM.pdf]

nature portfolio

Peer Review File

Jet-Induced Rainfall Seasonality and C4 Migration over East Asia

Corresponding Author: Dr Jiawei Da

This file contains all reviewer reports in order by version, followed by all author rebuttals in order by version.

Version 0:

Reviewer comments:

Reviewer #1

(Remarks to the Author)
This paper presents the reconstructed temperature and oxygen isotope of soil water and precipitation since the Late Pliocene based on isotopic analysis of calcium nodules in three red clay profiles on the Chinese Loess Plateau. The results reveal significant increases in both temperature and precipitation oxygen isotope across the Miocene and Pliocene boundary. By combining marine and terrestrial records with model results, the authors suggest that the seasonal variation in precipitation caused by the southward migration of the westerly jet, controlled by meridional temperature gradient controlled by the amplitude of obliquity variability, is the main driver for C4 vegetation expansion around 5 Ma. This study extends the co-evolution of westerly and monsoon from a seasonal scale to tectonic and orbital timescales, implying that different seasonal change in monsoon precipitation during the Miocene-Pliocene warm period has profound impact on the vegetation change. The reliable data and novel conclusions are suitable for possible publication in Nat. Commun. Some specific suggestions are as follows:
1.Lines 83-116 (Fig. 2): This figure mainly illustrates asynchronous timing of C4 expansion revealed by marine and terrestrial records in East Asia. The exact timing of each record should be clearly highlighted in these proxy records. It seems that the carbon isotope record of black carbon from the Lingtai profile doesn’t show a significant C4 expansion.Carbon isotope of carbonate nodules from several loess-red clay profiles (e.g., An et al., 2005) has been reported to infer the monsoon-induced vegetation change. These records should be briefly compared and commented to address the monsoon-vegetation coupling process.
2.Lines 169-172 (Fig. 3c and d): This sentence describes a temperature increase from an average of 12°C in the Late Miocene to 31°C at 5.1 Ma. First, the average temperature from 7 to 5.3 Ma seems to be around 15°C. Second, the range of variation should be described with an average temperature from 5.3 to 2.7 Ma, rather than a single highest temperature. Otherwise, pretty high temperature fluctuation (~19°C) is difficult to be explained by small-amplitude change in the meridional temperature gradient. I would suggest to describe the average temperature difference between late Miocene (7-5.3 Ma) and Pliocene (5.3-2.7 Ma).
3.Lines 200-231 (Fig. 4j): This figure mainly discusses the southward shift of the westerly winds and its impact on vegetation. The schematic diagram of the westerly position in Fig. 4j lacks of independent supporting evidence from geological records, e.g. Eolian records from the North Pacific Pacific can be compared to verify whether the westerly winds shifted southward from the Late Miocene to the Pleistocene.
4.Lines 392-396: The authors want to discuss the impact of vegetation change on human evolution, but they did provide direct evidence of vegetation change and human activities, making it difficult to clarify the impact of seasonal precipitation on human migration. More direct evidence should be provided to confirm the impact of seasonal precipitation on vegetation change and human activities.

Reviewer #2

(Remarks to the Author)
Review of „Jet-Induced Rainfall Seasonality and C4 Expansion over East Asia” by Da et al.

In this manuscript, a novel theory is proposed concerning the expansion of C4 plants in East Asia during the Late Miocene-Pliocene. The authors argue that this expansion was controlled by rainfall seasonality, which was in turn driven by the poleward-equatorward shifts of the subtropical westerly jet. The concept outlined is based on proxy data, including temperature, precipitation/soil water δ18O and aridity estimates derived from soil carbonates (SCs) originating from

Miocene-Pliocene red clays in East Asia, and C3/C4 vegetation composition estimated from δ13C of various terrestrial and marine sediment records. It also incorporates information on sea surface temperature gradients obtained from global ocean Uk37 records, and paleoclimate simulations for the mid-Pliocene using CESM2.

Overall, I have a very favourable impression of the manuscript. It is notable for its integration of novel proxies and the presentation of results from previously unpublished paleoclimate model runs. Furthermore, it introduces a concept that is logical and, for the most part, convincing. Nevertheless, in order to determine the manuscript's suitability for publication in Nature Communications, it is imperative to address the following unresolved issues, as they fundamentally impact the validity of the conclusions drawn. Below, I will address both methodological issues, including those related to the analytical part, and more conceptual issues.

Let's begin by considering some fundamental methodological issues that could potentially impact the conclusions of the manuscript under certain circumstances.

1) Firstly, the authors assume that the Chinese red clay sequences are continuous, which I consider to be unlikely; at most, they are quasi-continuous. I assume that the entire chronological model is based on this assumption. It would be beneficial for the authors to provide their insights on this matter.

2) Assuming the above concept is correct and the deposition ages can be accurately estimated based on magnetic susceptibility correlations (+tuning), the question arises as to when the SCs were formed in relation to the depositional ages. The authors assume that there may be a difference of 20-50 thousand years between the age of SCs and the age of deposition. Indeed, according to U-Th dating of SCs in Chinese loess-paleosol sequences, they post-date by ~40 ka the paleosol in which or under which they are found (Rowe and Maher, 2000). Further research indicates that this interval may be considerably longer (Újvári et al., 2024). Based on this work, in many cases carbonates in the same stratigraphic position are not of the same age, and the principle of superposition is often violated for SCs. Could you please clarify how this affects your conclusions? Have you tried to U-Pb-date your nodules? How is it possible to align and present carbonates from different red clay records on the age scale if their ages are so uncertain?

3) In relation to the subject of SC formation, it is evident, as the authors also point out, that due to their nature as cumulative soil profiles, it is regrettably not feasible to ascertain the depth of SC formation with respect to the paleosol surface. It is assumed that they formed at a depth of ~ 40-70 cm, and that the shallow-depth carbonates formed over obliquity minima were simply dissolved. In my opinion, the issue of overprinting is raised too late in the discussion; in fact, it is not mentioned until the last chapter, which I think is a problem. More fundamentally, the authors do not adequately address the fact that the reconstructed temperatures (T47) are significantly influenced not only by the seasonal shift in SC formation, but also by the depth of formation. In my opinion, the latter introduces an uncertainty of ~ 3-5 °C in the Δ47-based temperature reconstructions.

4) Some questions arise in relation to the oxygen stable isotope and clumped isotope measurements, such as why these measurements were performed in separate laboratories, sometimes using different methodologies? Were cross-checks performed to ensure data comparability between labs?

5) There are some issues that require clarification in relation to clumped isotope measurements. As stated in the text, the measurements were performed following the work of Burgener et al. (2016). This involved a three-point calibration using heated and temperature-equilibrated gases, and Δ47 values were corrected to the Absolute Reference Frame (ARF) using heated gas and CO2-water equilibration lines.

a) The description does not cover how to correct the negative background signal produced by secondary electrons. As the MAT 253 IRMS is not equipped with an m/z 47.5 Faraday cup, its signal cannot be used for baseline correction. This capability is only available on the 253 Plus model, which has a half-mass collector at m/z 47.5. I did not see any comments on this, nor on the PBL correction method used.

b) With regard to the NuCarb + Nu Perspective system, was the carbonate-based standardisation used? While the text alludes to this, it does not make the point explicitly.

c) It is essential to specify the number of replicates measured from the samples, the integration time used, etc. To enhance transparency, the measured data and their errors (including the oxygen isotope data) should be given in a separate table in the supporting information file. They should also provide the long-term SD values of carbonate standards. Currently, for instance, the T47 values are only shown in figures, which is not sufficient for evaluation.

6) Regarding the steady-state soil water isotope model, how representative is this model for natural soils or red clays? To what extent does it affect the data if the model assumptions are incorrect? For example, if evaporation affects not only the isotopic composition of shallow soil water but also that of deeper (>50 cm) zones, as found by Kelson et al. (2023)?

In summary, it is not being claimed that the issues mentioned above and in the line-by-line comments below mean that this material is fundamentally flawed. However, it is thought that they need to be answered in a reassuring manner so that the data in the manuscript can be accepted as robust results.

Line-by-line comments

Line 135, "Calcite formation temperatures…": More info is needed. In any case, this step may cumulate a fair amount of noise.

Line 178, Fig. 2 caption: Define MTG, the meridional temperature gradient.

Line 154, coherent T47: More or less true: sometimes there is a 10 °C difference between the values (see Fig. S3).

Line 155: Define the abbreviation of the Mio-Pliocene Boundary in parenthesis.

Line 160: It would be useful to include this average value in Figure 3e.

Line 224, T47-d18O coupling: How strong is this coupling? Could you quantify the correlation?

Line 228, "as high as > 10 °C…": Is this reasonable based on other proxies and modelling results?

Line 232-236: Another factor that significantly influences the recorded T47 is the depth of soil nodule formation. Virtually no information is available on this. Depending on the soil type, the difference in seasonal soil temperature between 0 and 100 cm can be roughly 3-5 degrees Celsius, maybe slightly more.

Line 295, "5.5-4 Ma": This is rather 5.0-4.5 Ma, in my view.

Line 306, NHG: For the average reader, the abbreviation NHG needs to be defined.
Line 355: Replace “ppm” with “mm”.
Lines 358-360: Assuming this is true, does virtually all variability in δ18Osw relate to seasonal changes during obliquity maxima? If so, is this variability still related to shifts in jet position, which basically follow obliquity minima-maxima? I may have lost track a little here.
Line 386: How do you know that? This is a continental sequence spanning millions of years. In my opinion, the chances of this red clay sequence being really continuous are pretty low.
Line 395: Have you taken images of the individual soil nodules? How do they look like? How do the internal structures of soil carbonates look like? Cathodoluminescence images? Any potential effects of diagenesis?
Line 405, offset: Again, this offset can be much larger, on the order of at least 100 ka.
Lines 411-412, “…32 and 22 nodule…selected”: Based on what criteria?
Line 438: Please assign a number to the article by Burgener et al. (2016). It is missing from the bibliography.

Figures
Figure 1, panel C: What do the different colours mean? Please clarify this in the caption, as some readers may not notice the color-coded sites on the map.
Figure 4: The figure caption and label do not match (figure: i-j, text: k-l).

Supporting information file
Line 26, Figure S1: Despite the statement in the caption, there are no blue boxes in this figure. Or am I just not seeing them?

References
Burgener, L., Huntington, K.W., Hoke, G.D., Schauer, A., Ringham, M.C., Latorre, C., Díaz, F.P., 2016. Variations in soil carbonate formation and seasonal bias over>4 km of relief in the western Andes (30∘S) revealed by clumped isotope thermometry. Earth Planet. Sci. Lett. 441, 188–199.
Kelson, J., Huth, T.E., Passey, B., Levin, N., et al. 2023. Triple oxygen isotope compositions of globally distributed soil carbonates record widespread evaporation of soil waters. Geochim. Cosmochim. Acta 355,138–160.
Rowe, P.J., Maher, B.A., 2000. ‘Cold’ stage formation of calcrete nodules in the Chinese Loess Plateau: evidence from U-series dating and stable isotope analysis. Palaeogeogr. Palaeoclim. Palaeoecol. 157, 109–125.
Újvári, G., Schneider, R., Stevens, T., Rinyu, L., et al. 2024. Absolute 230Th/U chronologies and Δ47 thermometry paleoclimate reconstruction from soil carbonates in Central Asian loess over the past 1 million years. Geochim. Cosmochim. Acta 386, 110–126.

Version 1:

Reviewer comments:

Reviewer #1

(Remarks to the Author)
I read through the revised paper and the response letter, and still have two minor suggestions. First, I did not see the arrow to mark the timing of the C4 vegetation expansion in Fig.2d-f. Second, in Figure 4, the authors added two eolian flux records from the North Pacific to infer the southward migration of the westerly jet during the warm Pliocene. I would suggest using the ratio of eolian flux (1208/885) to indicate the position shift of the westerly. it will be better to extend the flux ratio to 2-8 Ma, so that the shifting of the westerly during the Miocene-Pliocene and Pliocene-Pleistocene boundaries can be clearly inferred.

Reviewer #2

(Remarks to the Author)
Re-evaluation (R1) of „Jet-Induced Rainfall Seasonality and C4 Expansion over East Asia” by Da et al.

The authors have satisfactorily addressed most of my previous concerns, and the revised manuscript reflects notable improvements. Overall, I am largely satisfied with the revisions. However, one issue remains unresolved and continues to concern me: the clumped isotope measurements obtained using the Nu Perspective instrument.
Specifically, the number of replicate measurements is still very low (only 2–5), whereas a minimum of approximately 10 repeat measurements is generally required to achieve statistically robust results (see Fernandez et al., 2017). I therefore remain unconvinced that the reported Δ_{47} measurements can reliably produce temperature uncertainties as low as 3–4°C. Furthermore, for IAEA C1-2 and Merck standards, the reported stable isotope values ($\delta^{13}C$ and $\delta^{18}O$) deviate from the nominal values by several per mil, yet the Δ_{47} values are reproduced accurately. This discrepancy raises an important question: how can the clumped isotope measurements be so accurate when the corresponding stable isotope measurements show such offsets?

Ref.
Fernandez, A., Müller, I. A., Rodriguez-Sanz, L., van Dijk, J., Looser, N., Bernasconi, S. M. (2017). A reassessment of the precision of carbonate clumped isotope measurements: Implications for calibrations and paleoclimate reconstructions. Geochemistry, Geophysics, Geosystems, 18. https://doi.org/10.1002/2017GC007106

Open Access This Peer Review File is licensed under a Creative Commons Attribution 4.0 International License, which permits use, sharing, adaptation, distribution and reproduction in any medium or format, as long as you give appropriate credit to the original author(s) and the source, provide a link to the Creative Commons license, and indicate if changes were made.
In cases where reviewers are anonymous, credit should be given to 'Anonymous Referee' and the source.
The images or other third party material in this Peer Review File are included in the article's Creative Commons license, unless indicated otherwise in a credit line to the material. If material is not included in the article's Creative Commons license and your intended use is not permitted by statutory regulation or exceeds the permitted use, you will need to obtain permission directly from the copyright holder.
To view a copy of this license, visit https://creativecommons.org/licenses/by/4.0/

Author replies to reviewer comments on Da et al. "*Jet-Induced Rainfall Seasonality and C_4 Expansion over East Asia* " [#NCOMMS-25-105841-T]

The reviewers' comments are in blue; our replies are in black.

Reviewer #1 (Remarks to the Author):

This paper presents the reconstructed temperature and oxygen isotope of soil water and precipitation since the Late Pliocene based on isotopic analysis of calcium nodules in three red clay profiles on the Chinese Loess Plateau. The results reveal significant increases in both temperature and precipitation oxygen isotope across the Miocene and Pliocene boundary. By combining marine and terrestrial records with model results, the authors suggest that the seasonal variation in precipitation caused by the southward migration of the westerly jet, controlled by meridional temperature gradient controlled by the amplitude of obliquity variability, is the main driver for C4 vegetation expansion around 5 Ma. This study extends the co-evolution of westerly and monsoon from a seasonal scale to tectonic and orbital timescales, implying that different seasonal change in monsoon precipitation during the Miocene-Pliocene warm period has profound impact on the vegetation change. The reliable data and novel conclusions are suitable for possible publication in Nat. Commun. Some specific suggestions are as follows:

1.Lines 83-116 (Fig. 2): This figure mainly illustrates asynchronous timing of C4 expansion revealed by marine and terrestrial records in East Asia. The exact timing of each record should be clearly highlighted in these proxy records. It seems that the carbon isotope record of black carbon from the Lingtai profile doesn't show a significant C4 expansion. Carbon isotope of carbonate nodules from several loess-red clay profiles (e.g., An et al., 2005) has been reported to infer the monsoon-induced vegetation change. These records should be briefly compared and commented to address the monsoon-vegetation coupling process.

We added arrows to highlight the timing of C_4 expansion shown in each record.

The $\delta^{13}C$ of black carbon increased from ~22‰ during the Plio-Pleistocene boundary to as high as 15‰ at 0.9 Ma, indicating an increase in C_4 contribution from 25% to 60% according to the original publication[1].

The $\delta^{13}C$ of pedogenic carbonate records atmospheric CO_2, soil respiration, and the $\delta^{13}C$ of aboveground vegetation[2]. Previous studies have confirmed that the $\delta^{13}C$ of carbonate nodules in loess-red clay profiles are significantly affected by the $\delta^{13}C$ and concentration of atmospheric CO_2[3,4], thus reflecting regional aridity rather than the $\delta^{13}C$ of vegetation. As such, we chose not to include nodule-$\delta^{13}C$ data from An et al. (2005)[5] when discussing vegetation change. This issue has already been discussed in the original manuscript (Lines 63-68).

2.Lines 169-172 (Fig. 3c and d): This sentence describes a temperature increase from an average of 12°C in the Late Miocene to 31°C at 5.1 Ma. First, the average temperature from 7 to 5.3 Ma seems to be around 15°C. Second, the range of variation should be described with an average temperature from 5.3 to 2.7 Ma, rather than a single highest temperature. Otherwise, pretty high temperature fluctuation (~19°C) is difficult to be explained by small-amplitude change in the meridional temperature gradient. I would suggest to describe the average temperature difference between late Miocene (7-5.3 Ma) and Pliocene (5.3-2.7 Ma).

We apologize for the statistical error. However, it should be noted that we do not interpret the changes in clumped isotope temperature as mean annual temperature change, as the formation time of pedogenic carbonates show strong seasonality in response to changes in seasonal rainfall distributions. This has been discussed in the manuscript (Lines 218-233).

The statistics and the original sentence have been modified per reviewer's suggestion:

" *Overall, both $T_{\Delta 47}$ and $\delta^{18}O_{sw}$ are higher during the Pliocene (5.3-2.7 Ma), with mean values of 20°C (SD = 5°C, n = 19) and -8.9‰ (SD = 1.1‰, n = 139), respectively, compared to the late Miocene (7-5.3 Ma) with lower averages of 16°C (SD = 4°C, n = 15) and -9.7‰ (SD = 0.6‰, n = 105). Superimposed on this long-term trend are three pronounced peaks in both $T_{\Delta 47}$ and $\delta^{18}O_{sw}$. The highest peak occurred at 5.1 Ma spanning the Miocene-Pliocene boundary (MPB, Fig. 3), with values of 29 ± 4°C and -6.7 ± 0.7‰, accompanied by two smaller peaks at 6.2 Ma and 3.5 Ma.* " (Lines 158-163)

3.Lines 200-231 (Fig. 4j): This figure mainly discusses the southward shift of the westerly winds and its impact on vegetation. The schematic diagram of the westerly position in Fig. 4j lacks of independent supporting evidence from geological records, e.g. Eolian records from the North Pacific can be compared to verify whether the westerly winds shifted southward from the Late Miocene to the Pleistocene.

Dust flux records[6] from the North Pacific Ocean spanning ~4.5-2.5 Ma have been added to Figure 4 (show below). As discussed in our original manuscript, the southward shift of the westerly jet during the Plio-Pleistocene transition due to the Northern Hemisphere Glaciation led to major C_4 expansion across the North China Plain:

" *Subsequently, the onset and intensification of the Northern Hemisphere Glaciation (NHG) pushed the mean jet position further south, as suggested by increases in dust fluxes in the mid-latitude North Pacific Ocean[6] (Fig. 4f-g). Meanwhile, organic carbon $\delta^{13}C$ values increase (3.5-2.5 Ma) and then decrease in the G3 record (Fig. 2e), consistent with the migration of a C_4-rich biome across the North China Plain.* " (Lines 285-289).

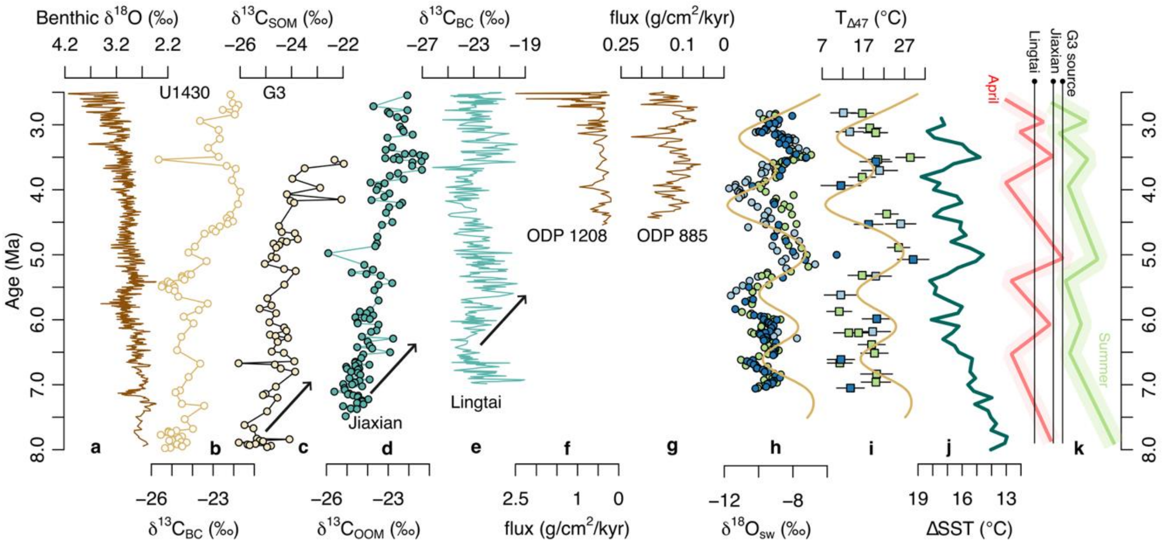

Figure R1 The late Miocene-Pliocene evolution of the westerly jet and regional climate over East Asia. (a) LR04 benthic $\delta^{18}O$ stack[7]. **(b-e)** Organic carbon $\delta^{13}C$ records across East Asia, including black carbon from U1430[8], sediment organic matter from the G3 drilling core[9], soil organic matter $\delta^{13}C$ from Jiaxian section[4], and black carbon from Lingtai section[1]. Note G3 data after 3.5 Ma are outside the axis range and therefore not shown here. **(f-g)** Dust fluxes at ODP sites 1208 and 885 from the mid-latitude North Pacific Ocean. **(h-i)** Reconstructed $\delta^{18}O_{sw}$ and $T_{\Delta 47}$ from this study. **(j)** Meridional sea surface temperature gradient (ΔSST) based on compiled SST records from tropical and high-latitude oceans (see *Methods*, Fig. S1). **(k)** Schematic diagram showing the meridional migration of the jet position during summer and April with respect to sampling locations of $\delta^{13}C$ records, drawn to be consistent with MTG changes inferred from ΔSST. The yellow curves in panels h-i show the 1.2-Myr obliquity amplitude modulation cycle.

4.Lines 392-396: The authors want to discuss the impact of vegetation change on human evolution, but they did provide direct evidence of vegetation change and human activities, making it difficult to clarify the impact of seasonal precipitation on human migration. More direct evidence should be provided to confirm the impact of seasonal precipitation on vegetation change and human activities.

We decided to remove the discussion of the impact of vegetation change on hominin evolution over northern China due to their complex interactions, which is beyond the scope of this study.

Reviewer #2 (Remarks to the Author):

Review of "Jet-Induced Rainfall Seasonality and C4 Expansion over East Asia" by Da et al.

In this manuscript, a novel theory is proposed concerning the expansion of C4 plants in East Asia during the Late Miocene-Pliocene. The authors argue that this expansion was controlled by rainfall seasonality, which was in turn driven by the poleward-equatorward shifts of the subtropical westerly jet. The concept outlined is based on proxy data, including temperature, precipitation/soil water δ18O and aridity estimates derived from soil carbonates (SCs) originating from Miocene-Pliocene red clays in East Asia, and C3/C4 vegetation composition estimated from δ13C of various terrestrial and marine sediment records. It also incorporates information on sea surface temperature gradients obtained from global ocean Uk37 records, and paleoclimate simulations for the mid-Pliocene using CESM2.

Overall, I have a very favorable impression of the manuscript. It is notable for its integration of novel proxies and the presentation of results from previously unpublished paleoclimate model runs. Furthermore, it introduces a concept that is logical and, for the most part, convincing. Nevertheless, in order to determine the manuscript's suitability for publication in Nature Communications, it is imperative to address the following unresolved issues, as they fundamentally impact the validity of the conclusions drawn. Below, I will address both methodological issues, including those related to the analytical part, and more conceptual issues.

Let's begin by considering some fundamental methodological issues that could potentially impact the conclusions of the manuscript under certain circumstances.

1) Firstly, the authors assume that the Chinese red clay sequences are continuous, which I consider to be unlikely; at most, they are quasi-continuous. I assume that the entire chronological model is based on this assumption. It would be beneficial for the authors to provide their insights on this matter.

The chronological framework of the Red Clay formation was established through magnetostratigraphy of multiple Red Clay sections across the Chinese Loess Plateau (CLP)[10]. The paleomagnetic records of all three sections contain every polarity subchron spanning 7.0-2.5 Ma. The relatively stable sedimentation rate across the late Miocene-Pliocene also suggest slow and continuous deposition[11–13]. Therefore, although it is hard to tell whether this formation was truly continuously deposited or not, the ages of each stratigraphic layer should be accurate at orbital to tectonic timescales, which is more than sufficient for the interpretation of our nodule-based records. We now provide more details on the chronology and deposition environment of the Red Clay formation:

" *The paleomagnetic records contain every polarity subchron spanning 7.0-2.5 Ma. The relatively stable sedimentation rate across the late Miocene-Pliocene also suggest slow and continuous deposition*[11–13]. " (Lines 381-383)

2) Assuming the above concept is correct and the deposition ages can be accurately estimated based on magnetic susceptibility correlations (+tuning), the question arises as to when the SCs were formed in relation to the depositional ages. The authors assume that there may be a difference of 20-50 thousand years between the age of SCs and the age of deposition. Indeed, according to U-Th dating of SCs in Chinese loess-paleosol sequences, they post-date by ~40 ka the paleosol in which or under which they are found (Rowe and Maher, 2000). Further research indicates that this interval may be considerably longer (Újvári et al., 2024). Based on this work, in many cases carbonates in the same stratigraphic position are not of the same age, and the principle of superposition is often violated for SCs. Could you please clarify how this affects your conclusions? Have you tried to U-Pb-date your nodules? How is it possible to align and present carbonates from different red clay records on the age scale if their ages are so uncertain?

To clarify, we are not attempting to resolve the 40 ky obliquity cycle but rather the 1.2 Myr obliquity amplitude modulation cycle. Several lines of evidence support that the age offset between pedogenic carbonates and the stratigraphy layer they are embedded within has minor effect on our interpretation of the nodule-based records:

1. As we mentioned in the manuscript (Lines 384-390), given the range of modern mean annual precipitation (MAP: 250-700 mm) across the CLP, the depth of pedogenic carbonate formation is unlikely to exceed 70 cm[14], which, using the sedimentation rates calculated from magnetostratigraphy, translate into a maximum of ~50 ky age offset. As it is generally accepted that MAP during the formation of Red Clay didn't exceed modern-day conditions[15], this age uncertainty is minor compared to the variations of our records discussed in the manuscript, which are at orbital-to-tectonic timescales (>0.1 Myr);
2. Published records of the $\delta^{13}C$ of pedogenic carbonates collected from multiple sections on the CLP show coherent trends with in-situ proxies such as magnetic susceptibility and the $\delta^{13}C$ of soil organic matter[4,5].

No, we haven't tried U-Pb dating on our nodules. U-Pb dating of pedogenic carbonates has been shown to work well on syn-pedogenic and early burial calcite cements[16], which provides useful constraints on the ages of deeper time paleosols. However, we do not anticipate U-Pb dating substantially improve our late Neogene chronology.

We now added a new sentence to discuss the age offset:

" *Based on the modern MAP across the CLP and the MAP-Bk depth relationship*[17]*, the depth of pedogenic carbonate formation is ~40-70 cm, which corresponds to ~20-50 ka offset given the sedimentation rate of the three Red Clay profiles. This age uncertainty is minor when interpreting long-term tectonic-scale records derived from pedogenic carbonates. Moreover, published pedogenic carbonate $\delta^{13}C$ records from multiple Red Clay sections co-varied with near-in-situ proxies, including magnetic susceptibility and the $\delta^{13}C$ of soil organic matter*[4,5]*, suggesting any potential lags due to translocation are minor at the timescale of interest here.* " (Lines 385-392)

3) In relation to the subject of SC formation, it is evident, as the authors also point out, that due to their nature as cumulative soil profiles, it is regrettably not feasible to ascertain the depth of SC formation with respect to the paleosol surface. It is assumed that they formed at a depth of ~ 40-70 cm, and that the shallow-depth carbonates formed over obliquity minima were simply dissolved. In my opinion, the issue of overprinting is raised too late in the discussion; in fact, it is not mentioned until the last chapter, which I think is a problem. More fundamentally, the authors do not adequately address the fact that the reconstructed temperatures (T47) are significantly influenced not only by the seasonal shift in SC formation, but also by the depth of formation. In my opinion, the latter introduces an uncertainty of ~3-5 °C in the Δ47-based temperature reconstructions.
We thank the reviewer for this thoughtful suggestion. To clarify, we didn't just assume that our nodule samples were formed at 40-70 cm depth, rather, they were estimated based on the likely ranges of mean annual precipitation (MAP) and the MAP-Bk relationship[14] (Lines 391-393).
We simulated depth-profile of monthly soil temperatures using an analytical heat diffusion model[18]:

$$T(z,t) = T_{avg} + A_0 \frac{\sin(\omega t - z/d)}{e^{z/d}}$$

Where the mean soil surface temperature (T_{avg} = 12°C) and amplitude of temperature variation (A_0=17°C) are based on modern monthly temperature observations across the CLP, ω is the radial frequency, and *d* is the damping depth, which is assumed to be 153 cm based on Quade et al. (2013)[19]. The differences between monthly modeled soil temperatures at 40 cm and 70 cm (the range of pedogenic carbonate formation depths considered reasonable based on rainfall estimates), are less than 3.2°C (Figure R2). In this respect, the effect of carbonate formation depth on clumped isotope temperature is minor compared to the large seasonal surface temperature variations (33°C on average).
We added Figure R2 (Fig. S3) as well as a new paragraph to discuss the effect of pedogenic carbonate formation depth on the clumped isotope temperature:

" *In addition to surface temperature, changes in pedogenic carbonate formation depth may contribute to $T_{\Delta 47}$ variations. Nonetheless, modeled monthly soil temperatures at potential carbonate formation depths vary by less than 3.3 ℃ based on modern observations across the CLP (Fig. S3), indicating the minor effect of carbonate formation depth on $T_{\Delta 47}$ compared to seasonal surface temperature variations.* " (Lines 154-160)

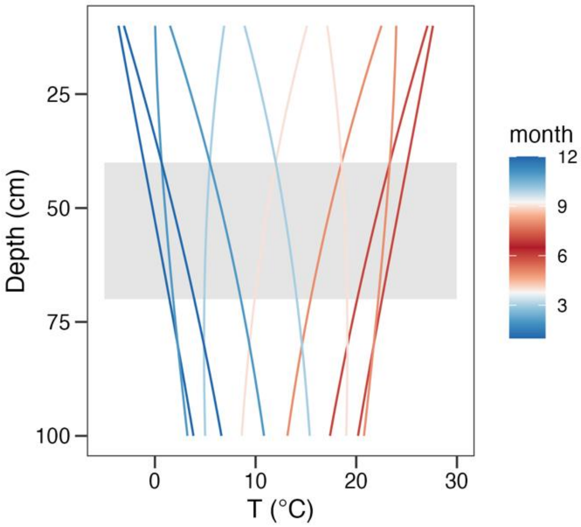

Figure R2 Modeled depth profile of monthly soil temperature variations based on modern surface temperature observations across the Chinese Loess Plateau (CLP, data from https://www.webmap.cn/), using a heat diffusion model[19]. The gray shade represents potential carbonate formation (Bk) depths based on modern mean annual precipitation (MAP) across the CLP and the MAP-Bk relationship[14].

4) Some questions arise in relation to the oxygen stable isotope and clumped isotope measurements, such as why these measurements were performed in separate laboratories, sometimes using different methodologies? Were cross-checks performed to ensure data comparability between labs?

Regarding the bulk isotopic analyses for $\delta^{18}O$ of pedogenic carbonates, they were measured in different labs because samples were collected and measured by different groups of people at different time. To ensure reproducibility, we measured four samples in both labs from Nanjing University and the University of Texas at Austin, and the $\delta^{18}O$ differences are within 0.1‰.

The clumped isotope measurements were performed on two different instruments in the same lab (Isolab, UW). For the 2019 analyses, samples were processed offline, and the measurements were made on a MAT 253 and standardized to the CDES (carbon dioxide equilibrium scale[20] using the methods of Burgener et al. (2016)[21] and Schauer et al. (2016)[22]. The wide range of Δ_{47} and bulk isotopic (δ_{47}) values of the equilibrated and heated CO_2 gases analyzed with this analytical session ensured robust statistical bracketing of the dataset. Carbonate standards including ETH1-4 were analyzed concurrently with the sample unknowns for quality control, but were not yet being analyzed with sufficient frequency given our large sample size needs for the method, nor did they represent sufficient compositional spread bracketing our samples, to enable robust I-CDES referencing. The lab then acquired newer instrumentation. The 2023 analyses were performed on a fully automated NuCarb-Nu Perspective system and standardized using carbonate standards ETH1, ETH2, ETH3, ETH4, Merck, IAEA-C1, and IAEA-C2 to the I-CDES (Intercarb carbon dioxide equilibrium scale) following D47Crunch[23]. An internal lab standard Coral was also analyzed for quality control. Equilibrated gases were not used in the calibration due to problems with the inlet system for introducing equilibrated CO_2 to the Nu Perspective at that time. Data reported in the CDES and I-CDES scales are theoretically equivalent because the carbonate standards used to anchor the I-CDES scale were themselves assigned accepted values via calibration to the CDES scale[24]. Consistent with this, the CDES-calibrated measured ETH standard

values from the MAT 253 dataset are in agreement with the accepted values for the materials[25] used to anchor the Nu-system data to the I-CDES scale. Full data and analytical details for all analyses are provided in the supplemental tables.

We now added more details regarding the measurements in the Method section:
" *To ensure measurements from different labs are comparable, four samples were measured for bulk isotopic composition in both labs, and the $\delta^{18}O$ differences are within 0.1‰.* " (Lines 421-423)
" *Δ_{47} values were calculated using established methods[26], with the exception that we used updated ^{17}O correction parameters[27], following recommendations of Daëron et al. (2016)[28] and Schauer et al. (2016)[22]. Samples analyzed using the Thermo MAT 253 IRMS were corrected to CDES-90°C acid values using CO_2 equilibrated at 4, 60 and 1000°C. Δ_{47} uncertainties for individual samples were given as one standard error (1σ) of replicate analyses, calculated using the long-term standard deviation of carbonate standard analyses, or sample replicates, whichever is larger. Samples analyzed using the fully automated NuCarb-Nu Perspective system were standardized to I-CDES[20] 90°C acid values using ETH1-4, IAEA-C1, IAEA-C2, and MERCK standards[25], and calculations following D47Crunch[23] pooled standardization with fully propagated Δ_{47} uncertainties. The I-CDES and CDES scales are mathematically identical and interchangeable[25], confirmed by agreement of ETH standard values from the MAT 253-CDES dataset with the accepted values[25] used to anchor the Nu-system data to the I-CDES scale (see Supplementary Tables). Clumped isotope temperature ($T_{\Delta 47}$) values reported here were calculated from mean Δ_{47} values using the Andersen et al. (2021)[29] calibration for samples digested at 90°C (no acid fractionation correction).* " (lines 450-466)

5) There are some issues that require clarification in relation to clumped isotope measurements. As stated in the text, the measurements were performed following the work of Burgener et al. (2016). This involved a three-point calibration using heated and temperature-equilibrated gases, and Δ47 values were corrected to the Absolute Reference Frame (ARF) using heated gas and CO2-water equilibration lines.
a) The description does not cover how to correct the negative background signal produced by secondary electrons. As the MAT 253 IRMS is not equipped with an m/z 47.5 Faraday cup, its signal cannot be used for baseline correction. This capability is only available on the 253 Plus model, which has a half-mass collector at m/z 47.5. I did not see any comments on this, nor on the PBL correction method used.
To address this comment, in the method section for the MAT 253, we have added the reference to Schauer et al. (2016)[22] (Line 472), which details the PBL background correction method that was performed, including all data processing scripts, alongside the reference to Burgener et al. (2016).

From Schauer et al. (2016): "*The pressure baseline (PBL)[10,24] was automatically measured using a modified background ISL script (detailed in the Supporting Information,*

Schauer_RCM_17OCorrectionClumped_sodatIISLScripts.docx) in the mass spectrometer software (Isodat version 3.0.88.1, Thermo Fisher Scientific; see next section) with the measurement made 0.08 kV left of peak center prior to each sample."

Additional measurement details are now supplied in the supplemental table metadata, including:

"Sample preparation: Sample digestion and purification was performed using a custom semi-automated off-line carbonate preparation device. For each analysis, 8-10 mg carbonate was digested for 10 minutes in 105 wt% (specific gravity = 1.904–1.970 g / mL) phosphoric acid at a constant temperature of 90 °C in a common acid bath. The evolved CO2 was cryogenically separated and passed with He carrier gas through an adsorption trap containing Porapak Q material (50/80 mesh) and silver wool before being sealed in a pyrex break seal and transferred to the IRMS.

Analysis: Samples were measured on a Thermo MAT 253 configured to measure m/z 44-49 inclusive (Burgener et al., 2016; Shauer et al., 2016). Briefly, background (pressure baseline) was measured off peak left before each sample. Peak centering was performed on m/z 44. Measurements were made at 16 V on m/z 44, with 90 sample-working gas comparisons, each 26 s, for a total integration time of 2340 s.

We performed 72 analyses of sample unknowns, 20 analyses of CO2 equilibrated at 4, 60 and 1000 °C to calibrate data to the carbon dioxide equilibrium scale (CDES, Dennis et al., 2011), and 23 analyses of carbonate standards for quality control. Carbonate standard analyses included 11 measurements of Intercarb standards (ETH1, ETH2, ETH3, ETH4; Bernasconi et al., 2021) and 12 analyses of intralaboratory carbonate standard (Coral, C2, C64) for quality control.

Data operations:

All scripts used for data processing are in Schauer et al. (2016) and use the Brand et al. (2010) correction parameters.

Values of $\delta^{18}O$, $\delta^{13}C$, and Δ_{47} were calculated from the mass spectrometer data, with $\delta^{18}O$ values referenced to NBS-18 and NBS-19 and $\delta^{13}C$ values referenced to NBS-19 and LSVEC.

Sample Δ_{47} data were corrected to the CDES90 reference frame (Dennis et al., 2011) using theoretical values of Δ_{47} for equilibrated CO_2 from Petersen et al. (2019) (reported in the table of Huntington and Petersen, 2023) after Wang et al. (2004).

Overall repeatability of Δ47 measurements was characterized using the standard deviation (SD, σ) of the long-term carbonate standards. Sample standard error (SE) was calculated by choosing whichever was larger: SD of the sample replicate measurements, or SD of the long-term carbonate standards.

Apparent equilibrium clumped isotope temperatures (TΔ_{47}) were derived from Δ_{47}-CDES90 values using the calibration of Anderson et al. (2021).

Data quality:

Repeatability of Δ_{47} (SD of all carbonate standards) 21.0 ppm

Repeatability of Δ_{47} (SD of intralaboratory carbonate standards) 18.1 ppm

Repeatability of Δ_{47} (SD of ETH1-4 carbonate standards) 24.8 ppm

Measured values of ETH2, ETH3, ETH4 are within 1 SD of the accepted values from Bernasconi et al. (2021), and ETH1 approaches 2SD of the accepted value."

b) With regard to the NuCarb + Nu Perspective system, was the carbonate-based standardization used? While the text alludes to this, it does not make the point explicitly.

The revisions to the Method section addressing reviewer comment 4 above now further clarify the calibration approach, making the point explicitly: " *Samples analyzed using the fully automated Nu Perspective system were standardized to I-CDES[20] 90°C acid values using ETH1-4, IAEA-C1, IAEA-C2, and MERCK standards[25], and calculations following D47Crunch pooled standardization with fully propagated Δ_{47} uncertainties[23].* " (Lines 461-463)

Additional details are now supplied in the NuPerspective supplementary table metadata, including:

> *"Sample preparation: Sample digestion and purification was performed using a NuCarb automated carbonate preparation device coupled to Nu Perspective IRMS. For each analysis, 1.2-1.5 mg carbonate was digested for 20 minutes to completion in 150 µL of 105 wt% (specific gravity = 1.90 g / mL) phosphoric acid at a constant temperature of 70 °C in an individual glass vial. The evolved CO2 was cryogenically separated and passed through an adsorption trap containing Porapak Q material (50/80 mesh) held at -30 °C and silver wool before being transferred to the IRMS.*
> *Analysis: Samples were measured on a Nu Perspective IRMS in 'cold finger' mode (e.g., Andersen et al., 2021). We performed 60 analyses of sample unknowns and 56 analyses of carbonate standards. Carbonate standard analyses included 50 measurements of Intercarb standards (ETH1, ETH2, ETH3, ETH4, MERCK, IAEAC1, or IAEAC2; Bernasconi et al., 2021) and 6 analyses of intralaboratory carbonate standard (Coral) for quality control. For each analysis, 30 sample-standard comparisons were measured for a total integration time of 600 s.*
> *Data operations:*

An R script called psi.R was used to take high frequency faraday-collector current measurements and convert them to isotope ratios and raw measured delta values and perform basic data quality screening. Psi_calibrate.py is used as an entry into D47crunch.py (Daëron, 2021).

Values of $\delta^{18}O$, $\delta^{13}C$, and Δ_{47} were calculated from the mass spectrometer data, and $\delta^{18}O$ and $\delta^{13}C$ values were referenced to the IAEA-C1 and ETH-4 carbonate standards using established methods (e.g., Huntington and Petersen, 2023).

Sample data were corrected to the I-CDES90 reference frame using accepted values of Δ_{47} for ETH1, ETH2, ETH3, ETH4, MERCK, IAEAC1, and IAEAC2 from Bernasconi et al. (2021).

Fully propagated analytical errors were calculated at the 95 % confidence interval (CI) using the pooled standardization method of Daëron (2021; D47crunch).

All scripts, data inputs and outputs are available for download in the accompanying zip file: Da-et-al_NuPerspectiveReport - session_230925E.zip.

Apparent equilibrium clumped isotope temperatures ($T\Delta_{47}$) were derived from Δ_{47}-ICDES90 values using the calibration of Anderson et al. (2021).

Data quality:

Repeatability of Δ_{47} (anchors) 19.7 ppm

Repeatability of Δ_{47} (unknowns) 22.3 ppm

Repeatability of Δ_{47} (all) 21.1 ppm"

c) It is essential to specify the number of replicates measured from the samples, the integration time used, etc. To enhance transparency, the measured data and their errors (including the oxygen isotope data) should be given in a separate table in the supporting information file. They should also provide the long-term SD values of carbonate standards. Currently, for instance, the T47 values are only shown in figures, which is not sufficient for evaluation.

We now provided the detailed measured data in the online Figshare database (https://doi.org/10.6084/m9.figshare.29137106), including the measured data (i.e., $\delta^{18}O$, Δ_{47}, and $\Delta'^{17}O$) and their errors, as well as the number of replicates.

For the clumped isotope data, we include summary tables as well as full replicate level data for samples and standards, for both the MAT 253 and Nu Perspective analyses. We also added more metadata to the clumped isotope supplemental data tables as noted in our responses to b) and c) above.

The supplementary tables and data are:

- Table S1. Clumped isotope data summary

- Table S2. NuPerspective analytical details and replicate data for all carbonate standards and sample unknowns
- Table S3. MAT 253 analytical details and replicate data for all carbonate standards, equilibrated/heated CO2, and sample unknowns, MAT253
- Da-et-al_NuPerspectiveReport - session_230925E.zip. Contains all scripts, data inputs and outputs.

These Supplementary Tables include the requested details about the analyses, including:

MAT 253 dataset

- n = 72 analyses of sample unknowns, 3-6 replicates per sample
- n = 20 analyses of equilibrated or heated CO_2 used as anchors in the CDES calibration
- n = 23 analyses of carbonate standards for quality control and to establish long-term repeatability.
- long-term repeatability of D47 is 18.1 ppm for intralaboratory carbonate standards Coral, C2 and C64, and 24.8 ppm for ETH1-4 (calculated from the residuals of carbonate standard values as SD = sqrt($\Sigma(x_i - \bar{x})^2 / (n - 1)$))
- The summary table metadata section now lists the integration time, which is 2340 seconds.

Nu-system dataset

- n = 60 analyses of sample unknowns, 3-6 replicates per sample
- n = 56 analyses of carbonate standards: 50 Intercarb carbonate standard measurements used to anchor the I-CDES reference frame, and 6 analyses of internal laboratory standard C2 for quality control
- long-term repeatability of D47 is 19.7 ppm for Intercarb standards (anchors), and 21.1 ppm overall (calculated using D47Crunch, Daëron 2021).
- The summary table now lists the integration time, which is 600 seconds.

6) Regarding the steady-state soil water isotope model, how representative is this model for natural soils or red clays?

We don't know for sure whether this model applies to the Red Clay formation, but it represents the current state of the science. In Kelson et al. (2023)[30], model-data comparison was made on the $\delta^{18}O$ and $\Delta'^{17}O$ of soil waters from Holocene inceptisols from Tibet, which yielded overall good agreement (Fig. S4 in that paper). Although this is the only work to date that targets the $\Delta'^{17}O$ of soil waters, the original Barnes and Allison model[31] that the Kelson (2023) model was built on, simulates the $\delta^{18}O$ and δD of soil waters that align with multiple lab column experiments[32], and has been widely applied to dryland soils. Together, these lines of evidence provide indirect support for applying this modeling framework to the Red Clay formation.

To what extent does it affect the data if the model assumptions are incorrect? For example, if evaporation affects not only the isotopic composition of shallow soil water but also that of deeper (>50 cm) zones, as found by Kelson et al. (2023)?

To clarify, the data presented in this study, including $T_{\Delta 47}$ and $\delta^{18}O$ and $\Delta'^{17}O$ of soil water, are entirely independent of this model (see detailed calculations in Method).

Substantial, deep evaporation would affect our interpretations. However, there are fairly well-understood physical limits on the depth to which evaporation can exert a significant effect. Specifically, evaporation rate is controlled by the water vapor pressure gradient. As the evaporation front migrates to deeper soils, this gradient decreases, which slows the evaporation rate.

The soil-water-isotope model used here[30] accounts for the effect of evaporation on soil water isotopes at all depths as a function soil depth, RH, air temperature, and evaporation rate. To minimize the potential bias from assumptions associated with the input parameters, we chose wide prior ranges (uniform distributions) for the environmental variables in the model, including relative humidity (RH=10-90%), $\delta^{18}O$ of rainfall ($\delta^{18}O_p$ = -30 - -5‰), evaporation rate (10^{-10} – 10^{-9} m/s), mean annual precipitation (normal distribution, mean = 500 mm, SD = 100mm), and mean annual surface temperature (normal distribution, mean = 10, SD = 5℃). These weak prior constraints allowed the model to fully explore reasonable environmental parameter space, yielding posterior estimates that are most consistent with the proxy data.

We now provide more details on the steady-state model and its applicability to red clay paleosols: " *To quantitatively constrain environmental variables based on proxy data, here we inverted for $\delta^{18}O_p$, mean annual surface temperature (MAST), mean annual precipitation (MAP), soil water evaporation rate, and relative humidity (RH), with a quasi-steady-state soil water isotope model[30,33,34] using a Markov Chain Monte Carlo approach conditioned on the proxy data ($\delta^{18}O_c$, $\Delta'^{17}O_c$, Δ_{47})[35]. The steady-state model quantifies the effect of evaporation on $\delta^{18}O_{sw}$ and $\Delta'^{17}O_{sw}$ as a function of soil depth, RH, MAST, and evaporation rate[30,31]. The analyses were performed in R version 4.4.2[36] using the rjags[37] and R2jags package[38]. To avoid biased interpretation of our proxy data, we set loose prior ranges for all the related environmental inputs, including RH (10-90%,), $\delta^{18}O_p$ (-25 - -5‰), evaporation rate (10^{-10} – 10^{-9} m/s), MAP (normal distribution, mean = 500 mm, SD = 100mm), and MAST (normal distribution, mean = 10, SD = 5 ℃), based on either theoretical constraints[30] or modern observations across the CLP. These weak prior constraints allowed the model to fully explore environmental parameter space and conservatively determine that $\delta^{18}O_p$ is likely the predominant control on $\delta^{18}O_{sw}$.* " (Lines 534-549)

In summary, it is not being claimed that the issues mentioned above and in the line-by-line comments below mean that this material is fundamentally flawed. However, it is thought that they need to be answered in a reassuring manner so that the data in the manuscript can be accepted as robust results.

Line-by-line comments

Line 135, "Calcite formation temperatures…": More info is needed. In any case, this step may cumulate a fair amount of noise.

We have expanded this sentence with more details:

" *Calcite formation temperatures used to calculate $\delta^{18}O_{sw}$ were obtained by interpolating $T_{\Delta 47}$ values within individual sections. Interpolation was restricted to intervals bracketed by adjacent $T_{\Delta 47}$, and the resulting temperature uncertainty was propagated into the $\delta^{18}O_{sw}$ estimates.* " (Lines 130-132)

Line 178, Fig. 2 caption: Define MTG, the meridional temperature gradient.

Done. (Line 93)

Line 154, coherent T47: More or less true: sometimes there is a 10 °C difference between the values (see Fig. S3).

We revised the original sentence to lower the tone:

" *The clumped isotope temperature ($T_{\Delta 47}$) and $\delta^{18}O_{sw}$ from these three sections on the CLP show generally coherent trends across the late Miocene - Pliocene (Fig. 3c-d).* " (Lines 150-152)

Line 155: Define the abbreviation of the Mio-Pliocene Boundary in parenthesis.

Done. (Line 162)

Line 160: It would be useful to include this average value in Figure 3e.

We added a vertical line to highlight the average value of $\Delta'^{17}O_{sw}$.

Line 224, T47-d18O coupling: How strong is this coupling? Could you quantify the correlation?

We added the following figure (Fig. R3) into the supplementary material (Fig. S4). We performed York linear regression[39] on paired $T_{\Delta 47}$ - $\delta^{18}O_{sw}$ data that account for uncertainties associated with both parameters. The regression result shows significant correlation with p-value less than 0.001.

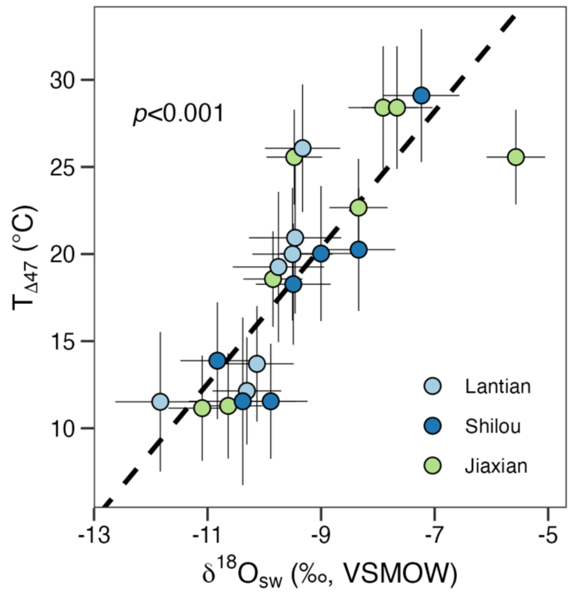

Fig. R3 Cross-plot of $T_{\Delta 47}$ vs. $\delta^{18}O_{sw}$, color-coded by different sections. The dashed line is the best fitting line based on York linear regression[33] that accounts for uncertainties associated with both parameters.

Line 228, "as high as > 10 °C…": Is this reasonable based on other proxies and modelling results?

As we discussed in the manuscript (copied below), this amplitude of variation is entirely possible after accounting for changes in the seasonality of pedogenic carbonate formation:

" *Moreover, our $T_{\Delta 47}$ records show large amplitude of variations (>10°C) across the MPB (Fig. 3c). For comparison, mean annual surface temperature (MAST) variability on the CLP across the Pleistocene glacial cycles is less than 10°C*[40]. *Given that regional climate variability is much less pronounced during the late Miocene-Pliocene periods*[15], *it is unlikely that changes in $T_{\Delta 47}$ are solely caused by MAST. Pedogenic carbonates tend to record seasonal climate signals rather than annual means*[41]. *In arid regions such as the CLP, pedogenic carbonate formation is primarily driven by rainfall events through its control over soil water chemistry*[42]. *Consequently, pedogenic carbonates grow after the rainy season under enhanced evaporation and reduced soil respiration*[43]. *During cold periods, CLP rainfall occurred during summer due to the lower latitude position of the westerly jet. Consequently, pedogenic carbonates grew during late summer and early fall, recording low summer-$\delta^{18}O_p$ and fall temperature. During warmer periods, an early onset of spring rain and intensive summer evaporation likely facilitate pedogenic carbonate growth during mid-summer, recording high spring-$\delta^{18}O_p$ and summer temperature. Modern surface temperature difference on the CLP between July and October can be as high as 17°C. Thus, the large $T_{\Delta 47}$ variability we observe is likely caused by seasonal shifts in soil carbonate formation superimposed on the long-term MAST changes.* " (Lines 218-233)

Line 232-236: Another factor that significantly influences the recorded T47 is the depth of soil nodule formation. Virtually no information is available on this. Depending on the soil type, the difference in seasonal soil temperature between 0 and 100 cm can be roughly 3-5 degrees Celsius, maybe slightly more.

Please see our reply to Comment 3.

Line 295, “5.5-4 Ma”: This is rather 5.0-4.5 Ma, in my view.
We modified the time to "5.3-4.0 Ma" following the description of the original publication[8]. (Line 283)

Line 306, NHG: For the average reader, the abbreviation NHG needs to be defined.
The abbreviation has been defined in the manuscript. (Line 285)

Line 355: Replace “ppm” with “mm”.
Corrected. (Line 343)

Lines 358-360: Assuming this is true, does virtually all variability in δ18Osw relate to seasonal changes during obliquity maxima? If so, is this variability still related to shifts in jet position, which basically follow obliquity minima-maxima? I may have lost track a little here.
Yes, we suggest that after obliquity maxima, increased meridional thermal gradient pushed the mean jet position towards lower latitude, leading to more rainfall during the summer, when enhanced evapotranspiration prevented soil water from reaching the depth of pedogenic carbonates formed during obliquity maxima.

Line 386: How do you know that? This is a continental sequence spanning millions of years. In my opinion, the chances of this red clay sequence being really continuous are pretty low.
Please see our reply to Comment 1 and 2.

Line 395: Have you taken images of the individual soil nodules? How do they look like? How do the internal structures of soil carbonates look like? Cathodoluminescence images? Any potential effects of diagenesis?
We examined the internal structure of individual nodules using SEM. As shown in Fig. R4, calcite mainly occurs as µm-scale, pore-filling cements associated with detrital quartz and clay minerals, suggesting pedogenic origin. Since the Red Clay formation has not been deeply buried, it is unlikely that the isotopic signals of these pedogenic carbonates were altered by burial diagenesis, consistent with the reasonable surface temperature recovered by clumped isotope analysis. Moreover, the coherent time-series variations in the stable isotope compositions of pedogenic carbonates across different sections, observed in both this study and previous work[4,44], further support the preservation of pristine isotopic signals.
We added Figure R4 in the supplementary file as well as more sentences in the manuscript to discuss diagenesis:
" *The internal structure of calcite nodules, revealed by Scanning electron microscope (SEM) imaging (Fig. S7), shows that calcite mainly occurs as secondary µm-scale, pore-filling cements associated with detrital quartz and clay minerals, suggesting pedogenic origin. Since the Red Clay formation has not been deeply buried, it is unlikely that the isotopic signals of these pedogenic*

carbonates were altered by burial diagenesis, consistent with the reasonable surface temperature recovered by clumped isotope analysis. Moreover, the coherent time-series variations in the stable isotope compositions of pedogenic carbonates across different sections, observed in both this study and previous work[4,44]*, further support the preservation of pristine isotopic signals.* " (Lines 405-412)

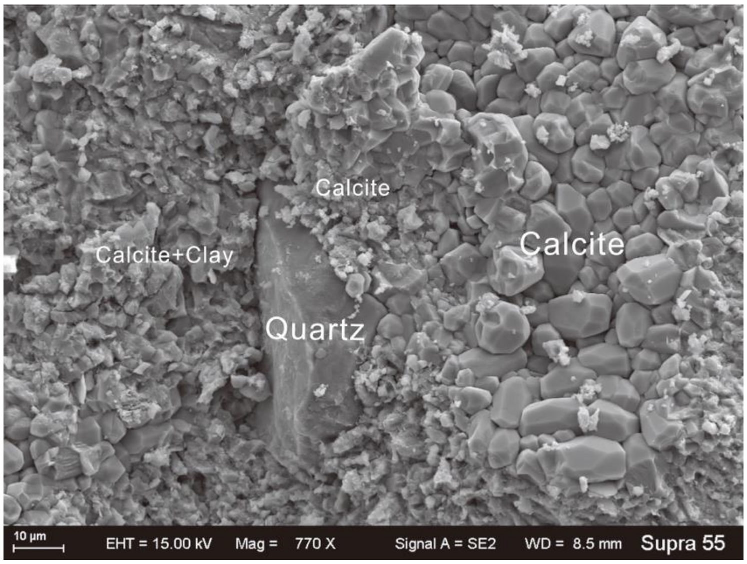

Figure R4 Scanning electron microscope (SEM) image of a calcite nodule from the Red Clay sequence at the Lantian section. Mineral phases shown in the image were confirmed by energy-dispersive X-ray spectroscopy (EDS).

Line 405, offset: Again, this offset can be much larger, on the order of at least 100 ka.
Please see our reply to Comment 1 and 2.

Lines 411-412, "…32 and 22 nodule…selected": Based on what criteria?
Owing to budget constraints and the time-intensive nature of both analyses, we selected 32 and 22 nodules that are evenly distributed across the full time interval. The primary criterion for selection was to maximize temporal coverage to capture long-term trends and variations.
We now expanded the original sentence to provide more context:
" *Owing to budget constraints and the time-intensive nature of both analyses, we selected 32 and 22 nodules for clumped and triple oxygen isotope analyses, respectively. The primary criterion for selection was to maximize temporal coverage to capture long-term trends and variations.* " (Lines 397-400)

Line 438: Please assign a number to the article by Burgener et al. (2016). It is missing from the bibliography.
Done. (Line 434)

Figures

Figure 1, panel C: What do the different colors mean? Please clarify this in the caption, as some readers may not notice the color-coded sites on the map.

We added the following sentence in the caption:

" *The monthly rainfall-$\delta^{18}O$ of the GNIP (Global Network of Isotopes in Precipitation) sites across the Asian monsoon region, with each site color-coded to match its location shown in panel a.* " (Lines 57-59)

Figure 4: The figure caption and label do not match (figure: i-j, text: k-l).

We thank the reviewer for this careful note. This has been corrected.

Supporting information file

Line 26, Figure S1: Despite the statement in the caption, there are no blue boxes in this figure. Or am I just not seeing them?

We are sorry for the confusion. To allow direction comparison of meridional SST gradients between the Pleistocene and Pliocene, we chose the same ODP sites for both time periods, hence no blue box. We have updated the sentence in the figure caption.

References

1. Zhou, B. *et al.* Late Pliocene–Pleistocene expansion of C4 vegetation in semiarid East Asia linked to increased burning. *Geology* **42**, 1067–1070 (2014).
2. Cerling, T. E. The stable isotopic composition of modern soil carbonate and its relationship to climate. *Earth & Planetary Science Letters* **71**, 229–240 (1984).
3. Da, J., Zhang, Y. G., Li, G. & Ji, J. Aridity-driven decoupling of δ13C between pedogenic carbonate and soil organic matter. *Geology* https://doi.org/10.1130/G47241.1 (2020) doi:10.1130/G47241.1.
4. Gallagher, T. M. *et al.* Regional patterns in Miocene-Pliocene aridity across the Chinese Loess Plateau revealed by high resolution records of paleosol carbonate and occluded organic matter. *Paleoceanography and Paleoclimatology* **n/a**, e2021PA004344 (2021).
5. An, Z. *et al.* Multiple expansions of C4 plant biomass in East Asia since 7 Ma coupled with strengthened monsoon circulation. *Geology* **33**, 705–708 (2005).
6. Abell, J. T., Winckler, G., Anderson, R. F. & Herbert, T. D. Poleward and weakened westerlies during Pliocene warmth. *Nature* **589**, 70–75 (2021).
7. Lisiecki, L. E. & Raymo, M. E. A Plio-Pleistocene stack of 57 globally distributed benthic δ 18 O records. *Paleoceanography* **20**, 1–17 (2005).
8. Shen, X. *et al.* Increased seasonality and aridity drove the C4 plant expansion in Central Asia since the Miocene–Pliocene boundary. *Earth and Planetary Science Letters* **502**, 74–83 (2018).
9. Lu, J. *et al.* The Early Pliocene global expansion of C4 grasslands: A new organic carbon-isotopic dataset from the north China plain. *Palaeogeography, Palaeoclimatology, Palaeoecology* 109454 (2019) doi:https://doi.org/10.1016/j.palaeo.2019.109454.

10. An, Z. *et al.* Late Cenozoic climate change in monsoon-arid Asia and global changes. in *Late Cenozoic Climate Change in Asia* 491–581 (Springer, 2014).

11. Ao, H. *et al.* Late Miocene–Pliocene Asian monsoon intensification linked to Antarctic ice-sheet growth. *Earth and Planetary Science Letters* **444**, 75–87 (2016).

12. Sun, D., Liu, D., Chen, M., An, Z. & John, S. Magnetostratigraphy and palaeoclimate of Red Clay sequences from Chinese Loess Plateau. *Sci. China Ser. D-Earth Sci.* **40**, 337–343 (1997).

13. Qiang, X. K., Li, Z.-X., Powell, C. M. & Zheng, H. B. Magnetostratigraphic record of the Late Miocene onset of the East Asian monsoon, and Pliocene uplift of northern Tibet. *Earth and Planetary Science Letters* **187**, 83–93 (2001).

14. Retallack, G. J. Pedogenic carbonate proxies for amount and seasonality of precipitation in paleosols. *Geology* **33**, 333–336 (2005).

15. Sun, Y., An, Z., Clemens, S. C., Bloemendal, J. & Vandenberghe, J. Seven million years of wind and precipitation variability on the Chinese Loess Plateau. *Earth and Planetary Science Letters* **297**, 525–535 (2010).

16. Aguirre Palafox, L. E. *et al.* U-Pb Geochronology of Paleosol Carbonate Cements by LA-ICP-MS: A Proof of Concept and Strategy for Dating the Terrestrial Record. *Geochemistry, Geophysics, Geosystems* **25**, e2024GC011488 (2024).

17. Retallack, G. J. Refining a pedogenic-carbonate CO2 paleobarometer to quantify a middle Miocene greenhouse spike. *Palaeogeography, Palaeoclimatology, Palaeoecology* **281**, 57–65 (2009).

18. Hillel, D. *Fundamentals of Soil Physics*. (Academic press, 2013).

19. Quade, J., Eiler, J., Daeron, M. & Achyuthan, H. The clumped isotope geothermometer in soil and paleosol carbonate. *Geochimica Et Cosmochimica Acta* **105**, 92–107 (2013).

20. Dennis, K. J., Affek, H. P., Passey, B. H., Schrag, D. P. & Eiler, J. M. Defining an absolute reference frame for 'clumped' isotope studies of CO2. *Geochimica et Cosmochimica Acta* **75**, 7117–7131 (2011).

21. Burgener, L. *et al.* Variations in soil carbonate formation and seasonal bias over >4 km of relief in the western Andes (30°S) revealed by clumped isotope thermometry. *Earth and Planetary Science Letters* **441**, 188–199 (2016).

22. Schauer, A. J., Kelson, J., Saenger, C. & Huntington, K. W. Choice of 17O correction affects clumped isotope (Δ47) values of CO2 measured with mass spectrometry. *Rapid Communications in Mass Spectrometry* **30**, 2607–2616 (2016).

23. Daëron, M. Full Propagation of Analytical Uncertainties in Δ47 Measurements. *Geochemistry, Geophysics, Geosystems* **22**, e2020GC009592 (2021).

24. Huntington, K. W. & Petersen, S. V. Frontiers of Carbonate Clumped Isotope Thermometry. *Annual Review of Earth and Planetary Sciences* **51**, 611–641 (2023).

25. Bernasconi, S. M. *et al.* InterCarb: A Community Effort to Improve Interlaboratory Standardization of the Carbonate Clumped Isotope Thermometer Using Carbonate Standards. *Geochemistry, Geophysics, Geosystems* **22**, e2020GC009588 (2021).

26. Huntington, K. W. *et al.* Methods and limitations of 'clumped' CO2 isotope (Δ47) analysis by gas-source isotope ratio mass spectrometry. *Journal of Mass Spectrometry* **44**, 1318–1329 (2009).

27. Brand, W. A., Assonov, S. S. & Coplen, T. B. Correction for the 17O interference in δ(13C) measurements when analyzing CO2 with stable isotope mass spectrometry (IUPAC Technical Report). *Pure and Applied Chemistry* **82**, 1719–1733 (2010).

28. Daëron, M., Blamart, D., Peral, M. & Affek, H. P. Absolute isotopic abundance ratios and the accuracy of Δ47 measurements. *Chemical Geology* **442**, 83–96 (2016).

29. Anderson, N. T. *et al.* A Unified Clumped Isotope Thermometer Calibration (0.5–1,100°C) Using Carbonate-Based Standardization. *Geophysical Research Letters* **48**, e2020GL092069 (2021).

30. Kelson, J. R. *et al.* Triple oxygen isotope compositions of globally distributed soil carbonates record widespread evaporation of soil waters. *Geochimica et Cosmochimica Acta* https://doi.org/10.1016/j.gca.2023.06.034 (2023) doi:10.1016/j.gca.2023.06.034.

31. Barnes, C. J. & Allison, G. B. The distribution of deuterium and 18O in dry soils: 1. Theory. *Journal of Hydrology* **60**, 141–156 (1983).

32. Allison, G. B., Barnes, C. J. & Hughes, M. W. The distribution of deuterium and 18O in dry soils 2. Experimental. *Journal of Hydrology* **64**, 377–397 (1983).

33. Fischer-Femal, B. J. & Bowen, G. J. Coupled carbon and oxygen isotope model for pedogenic carbonates. *Geochimica Et Cosmochimica Acta* **294**, 126–144 (2021).

34. Da, J., Bowen, G. J., Harper, D. T. & Huntington, K. Improving Paleosol-Based Atmospheric CO2 Reconstruction via Joint Proxy Inversion. *Paleoceanography and Paleoclimatology* **40**, e2025PA005317 (2025).

35. Lunn, D., Jackson, C., Best, N., Thomas, A. & Spiegelhalter, D. The BUGS book. *A practical introduction to Bayesian analysis, Chapman Hall, London* (2013).

36. R Core Team. *R: A Language and Environment for Statistical Computing*. (R Foundation for Statistical Computing, Vienna, Austria, 2024).

37. Plummer, M. rjags: Bayesian graphical models using MCMC (R package version 4-6, 2016). *Available at: https://CRAN. Rproject. org/package= rjags* (2018).

38. Su, Y.-S., Yajima, M., Su, M. Y.-S. & SystemRequirements, J. Package 'r2jags'. *R package version 0.03-08, URL http://CRAN. R-project. org/package= R2jags* (2015).

39. York, D., Evensen, N. M., Martínez, M. L. & De Basabe Delgado, J. Unified equations for the slope, intercept, and standard errors of the best straight line. *Am. J. Phys.* **72**, 367–375 (2004).

40. Lu, H. *et al.* Decoupled Land and Ocean Temperature Trends in the Early-Middle Pleistocene. *Geophysical Research Letters* **49**, e2022GL099520 (2022).

41. Kelson, J. R. *et al.* A proxy for all seasons? A synthesis of clumped isotope data from Holocene soil carbonates. *Quaternary Science Reviews* **234**, 106259 (2020).

42. Da, J., Li, G. K. & Ji, J. Seasonal changes in the formation time of pedogenic carbonates on the Chinese Loess Plateau during Quaternary glacial cycles. *Quaternary Science Reviews* **305**, 108008 (2023).

43. Breecker, D. O., Sharp, Z. D. & McFadden, L. D. Seasonal bias in the formation and stable isotopic composition of pedogenic carbonate in modern soils from central New Mexico, USA. *Geological Society of America Bulletin* **121**, 630–640 (2009).

44. Passey, B. H. *et al.* Strengthened East Asian summer monsoons during a period of high-latitude warmth? Isotopic evidence from Mio-Pliocene fossil mammals and soil carbonates from northern China. *Earth and Planetary Science Letters* **277**, 443–452 (2009).

Author replies to reviewer comments on Da et al. "*Jet-Induced Rainfall Seasonality and C4 Migration over East Asia* " [#NCOMMS-25-105841A]

The reviewers' comments are in blue; our replies are in black.

Reviewer #1 (Remarks to the Author):

I read through the revised paper and the response letter, and still have two minor suggestions. First, I did not see the arrow to mark the timing of the C4 vegetation expansion in Fig.2d-f.

We apologize for this overlook. The arrows have now been added to Fig. 2 (Figure R1 below) in the manuscript.

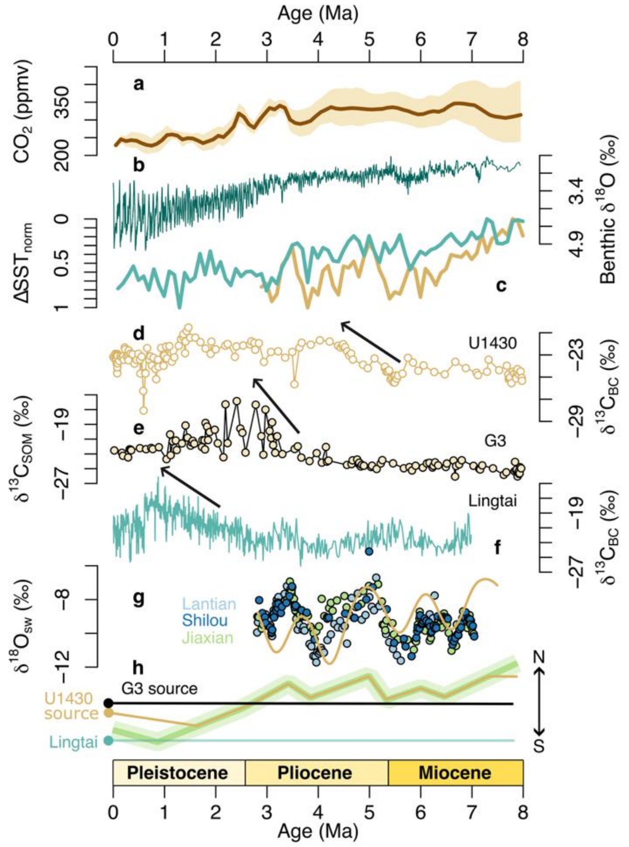

Figure R1 The long-term evolution of C_4 biomass over East Asia and global climate. (a) Atmospheric CO_2 estimates based on published proxy records via Bayesian inversion[1]. The dark line and shade show median and 68% credible interval, respectively. **(b)** LR04 benthic $\delta^{18}O$ stack[2]. **(c)** Normalized meridional sea surface temperature gradient (ΔSST_{norm}) based on the differences of compiled SST records from tropical and mid-latitude oceans (green curve) and from tropical and high-latitude oceans[3] (brown curve, see *Methods*, Fig. S1). **(d)** Black carbon $\delta^{13}C$ from IODP (Integrated Ocean Drilling Program) U1430 in the Japan Sea[4]. **(e)** Sediment organic matter $\delta^{13}C$ from the G3 drilling core on the North China Plain[5]. **(f)** Black carbon $\delta^{13}C$ from Lingtai section at southern CLP[6]. Arrows in panels d-f mark the timing of C_4 expansion. **(g)** Reconstructed $\delta^{18}O$ values of soil water ($\delta^{18}O_{sw}$) from this study, color-coded by sampling sites. **(h)** Schematic diagram showing the meridional migration of the summer position of the westerly jet stream, drawn to be consistent with meridional temperature gradient (MTG) changes inferred from benthic $\delta^{18}O$ and ΔSST_{norm}. The oscillations in CLP $\delta^{18}O_{sw}$ and the various organic carbon $\delta^{13}C$ records can be understood as responses to changes in rainfall seasonality controlled by the movement of the westerly-jet tracking summer rain band.

Second, in Figure 4, the authors added two eolian flux records from the North Pacific to infer the southward migration of the westerly jet during the warm Pliocene. I would suggest using the ratio of eolian flux (1208/885) to indicate the position shift of the westerly. it will be better to extend the flux ratio to 2-8 Ma, so that the shifting of the westerly during the Miocene-Pliocene and Pliocene-Pleistocene boundaries can be clearly inferred.

Following the reviewer's suggestion, the dust flux ratio of site 1208 and 885 has been used in lieu of dust flux records (Figure R2f). However, high-resolution dust flux records from ODP site 1208 and 885/886 only extend to 4.5 Ma[1]. Nonetheless, an earlier low-resolution dust flux record from ODP site 885/886[2] (Figure R2g) shows a decrease in dust flux towards the Miocene – Pliocene boundary and an increase towards the Pliocene – Pleistocene boundary. This pattern is broadly consistent with our interpretation of a more poleward jet position during the warm Pliocene, driven by a reduced meridional temperature gradient (Figures R2j-k, R1c, and R1h).

We have now added both records in Fig. 4 as well as the following sentences in the main text:

" *the effect of the MTG on jet position and strength has been documented by dust flux and provenance records from the North Pacific Ocean*[1–3] *(Fig. 4f-g), suggesting poleward and weakened westerly winds during the warm Pliocene compared with the late Miocene and Pleistocene glacial periods.* " (Lines 217-220)

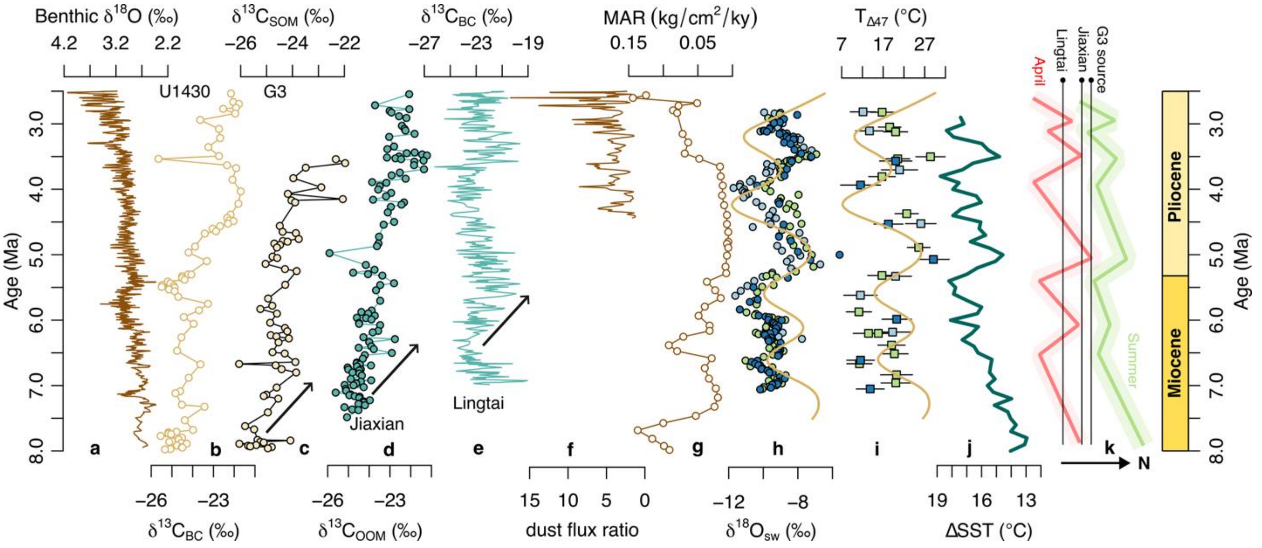

Figure R2 The late Miocene-Pliocene evolution of the westerly jet, C_4 biomass, and global climate. (a) LR04 benthic $\delta^{18}O$ stack[4]. **(b-e)** Organic carbon $\delta^{13}C$ records across East Asia, including black carbon from U1430[5], sediment organic matter from the G3 drilling core[6], soil organic matter $\delta^{13}C$ from Jiaxian section[7], and black carbon from Lingtai section[8]. Note G3 data after 3.5 Ma are outside the axis range and therefore not shown here. **(f)** Dust flux ratio between ODP sites 1208 and 885/886 from the mid-latitude North Pacific Ocean[1]. **(g)** Dust flux record from ODP site 885/886[2]. **(h-i)** Reconstructed $\delta^{18}O_{sw}$ and $T_{\Delta 47}$ from this study. **(j)** Meridional sea surface temperature gradient (ΔSST) based on compiled SST records from tropical and high-latitude oceans (see *Methods*, Fig. S1). **(k)** Schematic diagram showing the meridional migration of the jet position during summer and April with respect to sampling locations of $\delta^{13}C$ records, drawn to be consistent with MTG changes inferred from ΔSST. The yellow curves in panels f-h show the 1.2-Myr obliquity amplitude modulation cycle.

Reviewer #2 (Remarks to the Author):

The authors have satisfactorily addressed most of my previous concerns, and the revised manuscript reflects notable improvements. Overall, I am largely satisfied with the revisions. However, one issue remains unresolved and continues to concern me: the clumped isotope measurements obtained using the Nu Perspective instrument.

Specifically, the number of replicate measurements is still very low (only 2–5), whereas a minimum of approximately 10 repeat measurements is generally required to achieve statistically robust results (see Fernandez et al., 2017). I therefore remain unconvinced that the reported Δ_{47} measurements can reliably produce temperature uncertainties as low as 3–4°C.

The number of replicate measurements was constrained by the limited availability of homogeneous pedogenic carbonate nodule material. We agree that higher replication is desirable; however, replication levels of ~2–6 analyses per sample as we have reported here are common in studies of natural carbonates where material is limited. We therefore explicitly report the resulting uncertainties for each sample.

Analytical uncertainties have been fully propagated, incorporating both within-sample repeatability and uncertainty associated with standardization to the I-CDES90 reference frame using carbonate standards (Nu-system) or CDES90 reference frame using gas standards (MAT 253). The reported 3–4 °C uncertainties correspond to 1σ error. In the revised submission summary table, we additionally provide 95% confidence intervals for all temperature estimates.

We emphasize that the cited target of ~10 replicates is an ideal for maximizing precision, rather than a strict threshold for interpretability. At lower replication, uncertainty is correspondingly larger, but results remain meaningful when uncertainty is rigorously quantified and interpretations are framed accordingly.

Importantly, our interpretations do not rely on individual temperature estimates. Instead, they are based on consistent patterns across the dataset. This approach reduces sensitivity to any single measurement and ensures that conclusions are supported by reproducible trends rather than by highly precise estimates from individual samples.

In the revised manuscript, we added the following to note this point:

" *Although replication per sample is limited (n = 2–6) by material availability, analytical uncertainties are fully propagated, and interpretations are based on consistent patterns across the dataset rather than individual temperature estimates.* " (Lines 152-154)

Furthermore, for IAEA C1-2 and Merck standards, the reported stable isotope values ($\delta^{13}C$ and $\delta^{18}O$) deviate from the nominal values by several per mil, yet the Δ_{47} values are reproduced accurately. This discrepancy raises an important question: how can the clumped isotope measurements be so accurate when the corresponding stable isotope measurements show such offsets?

We thank the reviewer for raising this point. We had also identified and corrected this discrepancy during revision.

The offset in $\delta^{13}C$ and $\delta^{18}O$ values from the original Nu Perspective dataset arises from differences between our laboratory batch of MERCK carbonate (lot B1774559010) and the InterCarb MERCK material (lot B1164559515) reported in Bernasconi et al. (2021). Our $\delta^{13}C$ and $\delta^{18}O$ values are referenced to the IAEA-C1, ETH-4 and MERCK carbonate standards. Our original data reduction used published MERCK values; however, independent calibration of our MERCK batch against international $\delta^{13}C$ and $\delta^{18}O$ standards indicates slightly different $\delta^{13}C$ and $\delta^{18}O$ values (−45.12‰ and −13.84‰, respectively). We have updated the data reduction accordingly and included these details in the Supplemental information.

Importantly, the MERCK Δ_{47} values both before and after this bulk C and O recalibration agree with the accepted Δ_{47} value from Bernasconi et al. (2021). This reflects the fact that both batches of MERCK have the same clumping signature, and that Δ_{47} values are defined relative to a stochastic isotopic distribution and are therefore largely insensitive to small offsets in bulk $\delta^{13}C$ and $\delta^{18}O$ when data are normalized to the I-CDES90 reference frame using carbonate standards. Consistent with this, when MERCK is treated as an unknown and calibrated using the other InterCarb standards, the resulting Δ_{47} values agree with published InterCarb MERCK values. Thus, while the original bulk isotope values were offset by ~0.2–0.4‰, Δ_{47} values were unaffected (differences of 0.0000 to 0.0002 ‰). This calibration correction does not alter any interpretations. All values have been updated in the revised manuscript and Supplementary Information[9]. The Methods detailed in the Supplementary Information have been revised accordingly:

" *$\delta^{13}C$ and $\delta^{18}O$ values were referenced to the IAEA-C1, ETH-4 and MERCK carbonate standards using established methods (e.g., Huntington and Petersen, 2023). IsoLab's batch of MERCK (lot-B1774559010) has $\Delta 47$ values consistent with published InterCarb MERCK values and different $\delta^{13}C$ and $\delta^{18}O$ values (−45.12‰ and −13.84‰, respectively) than the original InterCarb MERCK material (lot-B1164559515). The reported isotopic values were independently calibrated against international $\delta^{13}C$ and $\delta^{18}O$ standards.* " (Supplemental Table S2)

References:

1. Abell, J. T., Winckler, G., Anderson, R. F. & Herbert, T. D. Poleward and weakened westerlies during Pliocene warmth. *Nature* **589**, 70–75 (2021).
2. Rea, D. K., Snoeckx, H. & Joseph, L. H. Late Cenozoic Eolian deposition in the North Pacific: Asian drying, Tibetan uplift, and cooling of the northern hemisphere. *Paleoceanography* **13**, 215–224 (1998).
3. Zhong, Y. *et al.* Humidification of Central Asia and equatorward shifts of westerly winds since the late Pliocene. *Commun Earth Environ* **3**, 1–9 (2022).
4. Lisiecki, L. E. & Raymo, M. E. A Plio-Pleistocene stack of 57 globally distributed benthic δ 18 O records. *Paleoceanography* **20**, 1–17 (2005).
5. Shen, X. *et al.* Increased seasonality and aridity drove the C4 plant expansion in Central Asia since the Miocene–Pliocene boundary. *Earth and Planetary Science Letters* **502**, 74–83 (2018).
6. Lu, J. *et al.* The Early Pliocene global expansion of C4 grasslands: A new organic carbon-isotopic dataset from the north China plain. *Palaeogeography, Palaeoclimatology, Palaeoecology* 109454 (2019) doi:https://doi.org/10.1016/j.palaeo.2019.109454.
7. Gallagher, T. M. *et al.* Regional patterns in Miocene-Pliocene aridity across the Chinese Loess Plateau revealed by high resolution records of paleosol carbonate and occluded organic matter. *Paleoceanography and Paleoclimatology* **n/a**, e2021PA004344 (2021).
8. Zhou, B. *et al.* Late Pliocene–Pleistocene expansion of C4 vegetation in semiarid East Asia linked to increased burning. *Geology* **42**, 1067–1070 (2014).
9. Da, J. Rainy spring delayed the late Neogene expansion of C4 biomass over East Asia. https://doi.org/10.6084/m9.figshare.29137106.v3 (2025) doi:10.6084/m9.figshare.29137106.v3.
